# Identification of risk areas for *Orobanche cumana* and *Phelipanche aegyptiaca* in China, based on the major host plant and CMIP6 climate scenarios

**DOI:** 10.1002/ece3.8824

**Published:** 2022-04-19

**Authors:** Lu Zhang, Xiaolei Cao, Zhaoqun Yao, Xue Dong, Meixiu Chen, Lifeng Xiao, Sifeng Zhao

**Affiliations:** ^1^ 70586 Xinjiang Production and Construction Corps Key Laboratory of Special Fruits and Vegetables Cultivation Physiology and Germplasm Resources Utilization Shihezi University Shihezi China; ^2^ 70586 Key Laboratory of Oasis Agricultural Pest Management and Plant Protection Resources Utilization Xinjiang Uygur Autonomous Region Shihezi University Shihezi China

**Keywords:** Broomarpe, climate change, environment variables, habitat shift, MaxEnt model, parasitic weeds, risk area

## Abstract

Parasitic broomrape of the genus *Orobanche* poses a formidable threat to producing many crops in Europe, Africa, and Asia. *Orobanche cumana* and *Phelipanche aegyptiaca* are two of China's most destructive root parasitic plants, causing extreme sunflower, tomato, melon, and tobacco damage. However, the potentially suitable areas of *O*. *cumana* and *P*. *aegyptiaca* in China have not been predicted, and little is known about the important environmental factors that affect their extension. Due to their invasiveness and economic importance, studying how climate change and host plants may affect broomrapes’ distribution is necessary. In the study, we first predicted the potentially suitable areas of the invasive weeds (*O*. *cumana* and *P. aegyptiaca*) and their susceptible host plants (*Helianthus annuus* and *Solanum lycopersicon*) using MaxEnt. Then, the risk zones and distribution shifts of two broomrapes under different climate conditions were identified by incorporating the distribution of their susceptible host plants. The results highlighted that the potential middle‐ and high‐risk zones for *O*. *cumana* and *P*. *aegyptiaca* amounted to 197.88 × 10^4^ km^2^ and 12.90 × 10^4^ km^2^, respectively. Notably, Xinjiang and Inner Mongolia were the highest‐risk areas within the distribution and establishment of *O*. *cumana* and *P*. *aegyptiaca*. Elevation and topsoil pH were the decisive factors for shaping *O*. *cumana* distribution; precipitation seasonality and annual precipitation were the dominant bioclimatic variables limiting the spread of *P*. *aegyptiaca*. The potentially suitable areas and risk zones of *O*. *cumana* would decrease significantly, and those of *P*. *aegyptiaca* would fluctuate slightly under future climate change scenarios. Overall, our study suggested that the two broomrapes’ risk zones will significantly northward to higher latitudes. The results will provide suggestions for preventing *O*. *cumana* and *P*. *aegyptiaca*.

## Graphical abstract

This study combined global climate change and host plants to examine the potential hazard status of *Orobanche cumana* and *Phelipanche aegyptiaca* in different climate change scenarios.

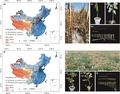

## 1 INTRODUCTION

Parasitic plants are ubiquitous species in ecosystems, including 292 genera and ca. 4,750 species (Nickrent, 2020). Some parasitic plants are considered pathogens due to their negative impact on host plants, such as *Orobanche* L., *Cuscuta* L., and *Striga* L. (Nickrent & Musselman, 2004). *Orobanche* L. (broomrape) was the typical obligate holoparasitic plant (Gevezova et al., 2012). They depend entirely on the host for survival, which reduces the host's ability to grow and may eventually cause the host's death (Musselman, 1980). Broomrapes include over 100 species and represent some of the most devastating parasitic plants, posing a significant challenge to agricultural production (Bari et al., 2019; Das et al., 2020; Wu & Qiang, 2006). In 2018, it was estimated that global annual losses owing to broomrapes damage were $1.3–2.6 billion (¥ 8.2–16.5 bn) (Ahmad et al., 2018). *Orobanche cumana* and *Phelipanche aegyptiaca* are widely distributed in northern China and cause catastrophic damage to tomatoes, sunflower, tobacco, legumes, carrot, and other crops (Wickett et al., 2011). The annual occurrence area only in Xinjiang was about 530–670 km^2^, causing economic losses of more than ¥ 0.5 bn (Yao et al., 2017). The damage induced to the crop by broomrape parasitism can range from 0% to 100%, which varies in each broomrape–host combination (Dhanapal et al., 1996). *Orobanche cumana* mainly parasitizes sunflower (*Helianthus annuus*). It was discovered for the first time parasitizing cultivated sunflower in Heilongjiang in 1959. Since then, *O*. *cumana* has spread over the sunflower cultivation area. In 2020, *O*. *cumana* occurred widely in the main sunflower producing area in China, and the occurrence rate was as high as 76.9%, resulting in some fields cannot continue to plant sunflowers, which has become the main factor restricting the sustainable development of sunflowers (Wu et al., 2020). Compared with *O*. *cumana*, *Phelipanche aegyptiaca* could parasitize more hosts (Kacan & Tursun, 2012; Parker, 2012). The tomato (*Lycopersicon esculentum*) is one of the significant vegetable crops in China that is highly vulnerable to infest by *P*. *aegyptiaca* (Tokasi et al., 2014). In 2013, the damaged area of *P*. *aegyptiaca* in tomato had reached about 70 km^2^ in Xinjiang province (about 10% of the total tomato planting area), and it spreads rapidly at a rate of 10%–20% every year (Chai et al., 2013; Zhang, Gan, et al., 2016). On average, tomato yield losses were between 30% and 80%, some of which were up to100% for being infected by broomrapes (Das et al., 2020).

It is challenging to control broomrapes because of their concealed underground for the major part of their life, lack of photosynthesis, and physical connection with the crops (Foy et al., 1989). The problem is exacerbated by the seed‐setting rate of broomrape is high and adaptable. Furthermore, this weed has strong invasive potential because the minute seeds can spread rapidly to distant fields in a short time. With climate change, there is possible for this weed to extend its distribution further and become an increasingly severe weed in certain areas. However, until now, no effective prevention measures have been developed for this weed (Libiaková et al., 2018). An alternative method is to carry out strict plant quarantine to prevent further dispersal in countries where *O*. *cumana* and *P*. *aegyptiaca* have not yet appeared (Hiroaki & Yukihiro, 2018). Therefore, it is vital to predict the potentially suitable areas of broomrapes and identify their critical environmental variables, which can alert the government to the potential risks on agriculture with future climate change and help them to propose positive coping strategies to prevent the spread of these weeds.

Environmental variables are biologically significant for determining the climatic niche of a species (Mohammadi et al., 2019; Qin, Liu, et al., 2017). Future climate change may have seriously affected global ecosystems and biotas by impacting species distributions (Hulme, 2017; Qin et al., 2017; Thomas et al., 2004). In the background of climate change, more and more attention has been concentrated on projecting the future distribution of species to mitigate the influences of future climate change on biodiversity (Ma & Sun, 2018). Species distribution models (SDMs) based on niche theory are a classical approach that uses species’ occurrence data combined with bioclimatic data to study the influences of climate change on species distribution (Schipper et al., 2014). Typical SDMs include CLIMEX, GARP, BIOCLIM, DOMAIN, and the maximum entropy (MaxEnt) algorithm (Byeon et al., 2018). Among which, the MaxEnt algorithm is better than other methods in determining potential species distributions based on environmental variables, either in terms of the quantity or in terms of the distribution scope of species records (Hernandez et al., 2010; Phillips & Dudík, 2008).

At present, there has been much research on forecasting the changing trend of potentially suitable areas in some species, such as invasive creatures, dominant forest species, precious and endangered species, but only a few studies have focused on parasitic plants (Ren et al., 2020; Wang et al., 2019; Zhang et al., 2016). Climate change can impact the distribution of parasitic plants by changing the geographical regions of host plants. The host plant distribution in conjunction with climate factors can enhance the prediction accuracy of the model (Berzitis et al., 2014). For example, the potential expansion of the co‐occurrence between pests and host species increased the harmful implications of fall armyworm (Zacarias, 2020). More recently, when analyzing factors affecting the distribution of emerald ash borer, it was found that adding host plant distributions in models could help with risk assessment (Dang et al., 2021). In parasitic plants, the availability of their main nutrient sources could be another determinant of broomrapes’ distribution under future climate. Thus, future control of the broomrapes should consider the influences of climate change and host plant distribution on broomrapes’ potentially suitable areas.

This research aimed to understand the potential risk status of *O*. *cumana* and *P*. *aegyptiaca* under future climate based on the host plant and climate change. Therefore, according to the occurrence records and environmental data, a niche model, MaxEnt, was used to examine important environmental variables affecting broomrapes’ distribution and predict their potentially suitable areas and risk zones under different climate scenarios. More specifically, the focus of this research was to: (1) determine the main environmental variables that are highly related with the potentially suitable areas of these two weeds; (2) predict the potentially suitable areas and risk zones of these two weeds under varying concentrations and years; (3) simulate and quantify the spatial pattern of shifts in the risk zones. These results can easily determine where is the most distributed or the most significant risk of two weeds and provide theoretical support and guide for the forewarning and prevention of *O*. *cumana* and *P*. *aegyptiaca*.

## 2 MATERIALS AND METHODS

### 2.1 Species study

*Orobanche* L., commonly known as broomrapes, are notorious global important invasive weeds. They are dicotyledonous holoparasitic flowering plants that attack a wide range of economically important crops in the Solanaceae, Asteraceae, Leguminous, and Cruciferae (Sauerborn et al., 2007). *Orobanche cumana* and *P*. *aegyptiaca* are the most problematic and damaging broomrape species in China. Botanically, *O*. *cumana* is characterized by unbranched inflorescence stems, while *P*. *aegyptiaca* is branched (Joel, 2009). The damage of *O*. *cumana* in sunflowers has lasted for nearly 100 years (Parker, 2009). In China, *O*. *cumana* was first discovered by a farmer in Zhaozhou County of Heilongjiang Province in 1959 (Shi & Zhao, 2020). Gradually, *O*. *cumana* spread widely to different sunflower planting areas of Inner Mongolia, Xinjiang, Heilongjiang, Shanxi, Jilin, Shaanxi, Hebei, Gansu, and Ningxia. *Orobanche cumana* had been a severe problem to limit sunflower productivity (Shi et al., 2015). *Phelipanche aegyptiaca* is a highly troublesome parasitic weed in the tomato field (Hershenhorn et al., 2009), and which was first recorded in Aksu of Xinjiang in 1953 (Zhang & Jiang, 1994). Since then, *P*. *aegyptiaca* has become the major problem in tomatoes and can totally destroy tomatoes under heavy infestation in Xinjiang.

### 2.2 Occurrence records collection and screening

**FIGURE 1:**
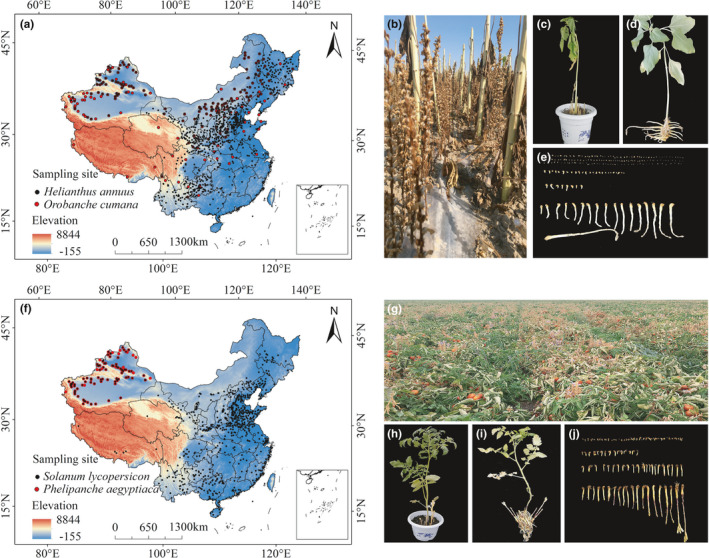
Distribution data of *Orobanche cumana* and *Phelipanche aegyptiaca* in China. (a) Distribution records of *O*. *cumana* and *Helianthus annuus* L. in China; (b) *O*. *cumana* is harmful to sunflower in the field; (c) *O*.*cumana* is harmful to sunflower in the pot experiment; (d) parasitism of *O*. *cumana* on sunflower root; (e) *O*. *cumana*. (f) Distribution records of *P*. *aegyptiaca* and *Solanum lycopersicon* in China; (g) *P*. *aegyptiaca* is harmful to tomato in the field; (h) *P*. *aegyptiaca* is harmful to tomato in the pot experiment; (i) parasitism of *P*. *aegyptiaca* on tomato root; (j) *P*. *aegyptiaca*

The current distribution records for two weeds and their host plant were obtained from among the following aspects: (1) online databases: the Global Biodiversity Information Facility (GBIF, https://www.gbif.org/), Chinese Virtual Herbarium (CVH, https://www.cvh.ac.cn/), Plant Photo Bank of China (PPBC, http://ppbc.iplant.cn/), and the National Specimen Information Infrastructure (NSII, http://www.nsii.org.cn/); (2) scientific publications: published articles, books, and reports (Appendix S1); and (3) field surveys: The information about the time, location, and host plants were recorded by field surveying in the *Orobanche* spp. the region in Xinjiang. In addition, the longitude and latitude were recorded using Google Earth (http://ditu.google.cn/). To minimize the error caused by the clustering effect, ArcGIS10.2 set up a 5 km × 5 km grid to ensure that there was only one distribution point in each grid. Finally, 190 data of *O*. *cumana* and 93 records of *P*. *aegyptiaca* were obtained for the modeling in China, and 837 data of *H*. *annuus* and 636 records of *S*. *lycopersicum* were obtained for the modeling. All current data were sorted by species name, longitude, and latitude and stored in a CSV format (Figure 1).

### 2.3 Environmental variables

A total of 34 environmental variables were screened that may impact the distribution of broomrapes and host plants (Table S1). The 19 bioclimatic factors were gained from WorldClim Global Climate Database (http://www.worldclim.org/) (Hijmans et al., 2010). The 14 soil factors were downloaded from China's soil map based on the harmonized world soil database (HWSD) (v1.1) (2009) (http://westdc.westgis.ac.cn) (Fischer et al., 2008). One topographic variable represented by elevation was downloaded from the World Soil Database (http://www.fao.org/faostat/en/#data.) (New et al., 1920).

Future climate scenarios were generated based on downscaled global climate model (GCM) data from CMIP6 (IPPC Sixth Assessment). Due to the climatic stronger warming, the CMIP6 model differs from CMIP5; the CMIP6 model has higher climate sensitivity and has new specifications in concentration, emission, and socio‐economic development (Ramasamy et al., 2021). In the sixth IPCC report, a new set of scenarios‐Shared Socioeconomic Pathways (SSPs) have been chosen to drive climate models for CMIP6. In precise, a set of scenarios were selected to support a series of different end‐of‐century climate change results. These scenarios were called SSP126, SSP245, SSP460, and SSP585, each of which represents the distinct probable future greenhouse gas concentration, thus making this set of scenarios ideal for exploring various warming pathways (Gidden et al., 2018). The extreme weather variations may greatly influence the future distribution pattern and its temporal and spatial variations for *O*. *cumana* and *P*. *aegyptiaca*. Therefore, SSP126 (the stringent mitigation greenhouse gas concentration scenario), SSP245 (the moderate greenhouse gas concentration scenario), and SSP585 (the scenario with very high greenhouse gas concentration) for the years 2050 (average for the years 2041–2060) and 2090 (average for the years 2081–2100) were chosen to predict future potentially suitable areas. In summary, the BCC‐CSM1.1 climate change modeling data under six climate change scenario/year combinations in the current study: SSP126‐2050, SSP126‐2090, SSP245‐2050, SSP245‐2090, SSP585‐2050, and SSP585‐2090. The climate prediction data were derived from the World Climate Database (http://www.worldclim.org/) (Hijmans et al., 2010).

Multicollinearity among climate factors could block the analysis of species–environment relationships. To avoid the influences of strong correlation environment factor on the projection outcomes, Principal component analysis (PCA), Spearman correlation analysis, and variance inflation factor (VIF) analysis of 34 environmental variables were tested utilizing the R x64 4.0.2 software (Wickham, 2009; Zhao et al., 2021). The environmental variables with absolute values of the Spearman correlation coefficient of less than 0.8 correlated ecological physiology and could be used for modeling (Qin et al., 2019). Then, the VIF analysis was performed on all variables, and the environmental factors with a VIF <10 were selected (Ye et al., 2020). Finally, in 34 variables, 14 and 10 factors were used for the *O*. *cumana* and *P*. *aegyptiaca* further modeling, respectively. Similarly, 11 and 12 factors were chosen for the *H*. *annuus* and *S*. *lycopersicum*, respectively (Table 1, Table S2).

**TABLE 1:** Percentage contributions and permutation importance of the environmental factors for *O*. *cumana* and *P*. *aegyptiaca*

Variable	Description	*O. cumana*		*P. aegyptiaca*	
		Percent contribution	Permutation importance	Percent contribution	Permutation importance
ELEV	Elevation	24.8	28.6	9.6	21.6
T_PH_H_2_O	Topsoil pH (H_2_O)	16.7	2.8	╳	╳
T_CEC_SOIL	Topsoil CEC (soil)	11.3	0.3	╳	╳
Bio19	Precipitation of Coldest Quarter	9.8	9.4	╳	╳
Bio1	Annual Mean Temperature	8.1	5.6	╳	╳
Bio7	Temperature Annual Range	6.0	4.6	4.0	1.6
Bio6	Min Temperature of Coldest Month	5.8	5.7	0.6	0.9
Bio18	Precipitation of Warmest Quarter	4.6	9.3	╳	╳
Bio11	Mean Temperature of Coldest Quarter	3.0	4.0	╳	╳
T_SILT	Topsoil Silt Fraction	3.0	5.7	╳	╳
Bio5	Max Temperature	2.6	7.7	0.7	0.8
T_TEB	Topsoil TEB	2.0	4.1	╳	╳
Bio12	Annual Precipitation	1.5	11.8	31.5	56.8
drainage	Soil Drainage Conditions	1.0	0.3	╳	╳
Bio15	Precipitation Seasonality	╳	╳	34.6	13
T_ESP	Topsoil Sodicity	╳	╳	7.1	2.3
T_BS	Topsoil Base Saturation	╳	╳	6.4	1.8
Bio2	Mean Diurnal Range	╳	╳	3.6	0.6
T_ECE	Topsoil Salinity (Elco)	╳	╳	1.8	0.5

### 2.4 MaxEnt modeling approach, optimization, and evaluation

In this research, the potential geographical distribution of broomrapes (*O*. *cumana* and *P*. *aegyptiaca*) and host plants (*H*. *annuus* and *S*. *lycopersicum*) under current and various future climate conditions were carried out in MaxEnt (maxent 3.4.1; http://www.cs.princeton.edu/wschapire/maxent) with presence‐only data. For each species, the training data were 75% of the sample distribution points chosen randomly, and the testing data were the remaining 25% of the sample points. The MaxEnt was run with a maximum number of iterations of 10,000, the 10^−5^ default convergence threshold, and 10 replicates under bootstrap run type to guarantee the model's accuracy (Wang et al., 2017). To ensure that the possibility of these species’ distribution appeared close to normal, the package “ENMeval” was used in R x64 4.0.2 (http://cran.us.r‐project.org) to optimize the MaxEnt model (Muscarella et al., 2015). The regulatory multiplier (RM) set ranged from 0.5 to 8, with increments of 0.5, for 16 values. The MaxEnt model includes five types of features: linear (L), quadratic (Q), hinge (H), product (P), and threshold (T) (Morales et al., 2017). In this research, we selected nine parameter combinations (FCs): L, LQ, H, LQH, LQHP, LQHPT, QHP, QHPT, and HPT. Finally, the ENMeval data package was employed to test the above 144 parameter combinations. The “checkerboard2” method was used to check the complexity and fit of the model by computing the Akaike Information Criterion coefficient (AICc), the difference between the training AUC and the testing AUC (avg.diff.AUC), and the 10% training omission rate (OR_10_). The lowest delta AICc values were chosen to establish the final MaxEnt models. AICc was the criteria to judge the accuracy of the model and when the model had the lowest AICc scores (delta. AICc =0), which was called the optimal model (Velasco & Gonzalez‐Salazar, 2019). Thus, RM = 4 and FC = QHP were selected for *O*. *cumana* in the ultimate software configuration. For *P*. *aegyptiaca*, RM = 1.5 and FC = LQH were chosen. Similarly, for the host plants, the model settings of RM = 1 and FC = HPT, RM = 1.5 and FC = QHPT significantly reduced the overfitting of the MaxEnt model, which is the best setting of model for *H*. *annuus* and *S*. *lycopersicum*, respectively (Figure S1). After the optimization was completed, the best combinations were utilized to model the potential habitats of broomrapes in different scenarios.

Using the spatial analysis module in ArcGIS10.4.1 to superimpose the suitable areas of broomrape and its host plant, the potentially suitable areas of broomrape based on host plants in China were final obtained, which was the potential risk zones of broomrape. The logistic format of MaxEnt output was applied in our study to assess the possibility of existence (values varied from 0 to 1) and the ultimate suitability map was divided into four classes: 0–0.1, unsuitable survival areas (no risk); 0.1–0.3, low suitability (low risk); 0.3–0.5, moderate suitability (medium risk); and 0.5–1, high suitability (high risk). The outputs were converted to raster format and areas were computed using the ArcMap tool in ArcGIS 10.4.1 for further analysis.

Jackknife testing was applied to assess the importance of variables. The relative importance of a single factor was evaluated by using default estimates of percentage contributions (Elith et al., 2015). Receiver operating characteristic (ROC) analysis was applied to assess the performance of the model, and the area under the ROC curve (AUC) was applied as an excellent index to provide the accuracy estimates (Katz & Zellmer, 2018; Peterson et al., 2017; Vanagas, 2004). Usually, AUC values were between 0 and 1. AUC < 0.5 represents a model that predicts worse than random and rarely occurs in reality, while AUC > 0.75 describes a model with a fair ability to discriminate (Ferrier, 2000). The closer the AUC is to 1, the better the prediction ability is (Yi et al., 2016). According to the AUC, prediction ability is classified as failing (0.5–0.6), poor (0.6–0.7), fair (0.7–0.8), good (0.8–0.9), or excellent (0.9–1) (Swets, 1988).

### 2.5 Analysis of spatial pattern changes

The spatial units with a species presence possibility value ≥0.3 was regarded as a suitable area for the species, and the value <0.3 was considered an unsuitable area. According to the (0,1) matrix table, the changes in the spatial pattern of the potential risk zones of two weeds under the different climate conditions were further analyzed. The area changes in the future were computed based on the present risk zones of the species. Four types of changes in the risk zones for species were defined: matrix values 0 → 1 were newly expanded suitable areas, 1 → 0 were lost suitable areas, 1 → 1 were reserved suitable areas, and 0 → 0 were unsuitable areas (Ye et al., 2020). Lastly, the matrix change value was loaded into ArcGIS 10.4.1 to realize the visualization of the spatial pattern change in different periods.

### 2.6 Centroids shifts

The changes in risk zones were computed, and the core distributional were compared for current and future risk zones by utilizing the SDM Toolbox (an ArcGIS tool) in ArcGIS10.4.1 (Brown, 2014). The focus of this analysis was to summarize the core distribution shifts in the risk zones of these two weeds. This was achieved by reducing the species’ risk zones to a center point and establishing a vector particle that reflected the magnitude and direction of the predicted changes through time. Finally, the shifts in species’ risk zones were checked by tracking how the core distributional changed with different SDMs, and the “dists” function in R x64 4.0.2 was used to calculate the centroid migration distance of the risk zones in latitude and longitude coordinates (Zhang et al., 2018).

## 3 RESULTS

### 3.1 Species distribution model performance

According to occurrences record of species and environmental data, we obtained potential distribution maps where broomrapes and host plants might occur. Models for the broomrapes and host plants performed better than random with the given series of training and test data. The average values of training AUC and testing AUC for all models were greater than 0.8, suggesting that these models were accurate for all studied species predictions and could be reliably used to simulate the relationship between diversity patterns and climatic variables (Figure S2).

### 3.2 Important Environmental variables

The analysis of single‐factor contributions indicated that elevation (ELEV, 24.8%), topsoil pH (T_PH_H_2_O, 16.7%), topsoil salinity (T_CEC_SOIL, 11.3%), precipitation of the coldest quarter (Bio19, 9.8%), annual mean temperature (Bio1, 8.1%), annual temperature range (Bio7, 6.0%), and minimum temperature of the coldest month (Bio6, 5.8%) made the significant contributions to the prediction model for *O*. *cumana*, the cumulative contribution value was reached up to 86.3% (Table 1). Among the *P*. *aegyptiaca* environmental variables selected, the important factors were precipitation seasonality (Bio15, 34.6%), followed by annual precipitation (Bio12, 31.5%), and elevation (ELEV, 9.6%). These three factors were the strongest predictor of *P*. *aegyptiaca* occurrence and contributed 75.7%. The topsoil sodicity (T_ESP, 7.1%) and topsoil base saturation (T_BS, 6.4%) were also found to contribute to the *P*. *aegyptiaca* prediction model (Table 1).

Using the response curve (Figure S3), we gained the thresholds (highly suitable possibility >0.5) for the important environmental factors. The response curves of the species prediction model of *O*. *cumana* indicated that the species of the high possibility of existence in areas with annual mean temperatures (Bio1) ranging from 3.6 to 12.8°C, the minimum temperature of the coldest month (Bio6) ranging from −20 to −4°C, an annual temperature range (Bio7) ranging from 41.9 to 66.7°C, precipitation of the coldest quarter (Bio19) ranging from 3.5 to 29.8 mm, elevation (ELEV) ranging from 250 to 1,448 m, topsoil salinity (T_CEC_SOIL) ranging from −7.6 to 4.9 cmol/kg, and topsoil pH (T_PH_H_2_O) ranging from 7.3 to 9. Similarly, there was a high possibility of *P*. *aegyptiaca* in regions with annual precipitation (Bio12) of 25.3–175 mm, precipitation seasonality (Bio15) of 11.6–62, elevation (ELEV) of 779–1,608 m, and topsoil sodicity (T_ESP) of 4–62.1%. Topsoil base saturation (T_BS) indicated no strong relation to highly suitable distribution for *P*. *aegyptiaca*.

### 3.3 Potential geographical distribution under current climate conditions

**FIGURE 2:**
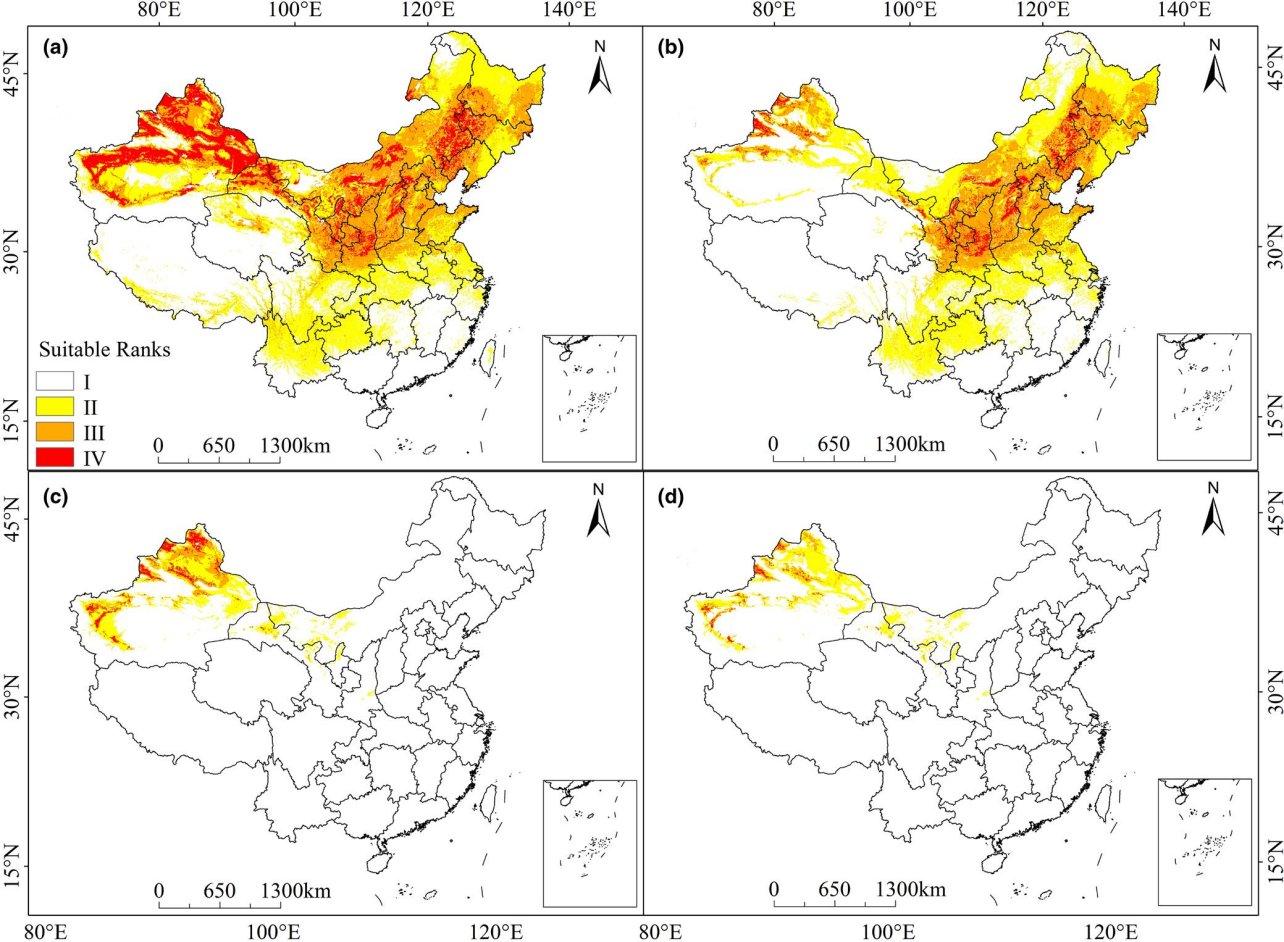
Current potentially suitable areas and risk zones of *Orobanche cumana* (a, b) and *Phelipanche aegyptiaca* (c, d) based on MaxEnt. (a) Potentially suitable areas of *O*. *cumana*; (b) Potentially risk zones of *O*. *cumana* based on *Helianthus annuus* L.; (c) Potentially suitable areas of *P*. *aegyptiaca*; (d) Potentially risk zones of *P*. *aegyptiaca* based on *Solanum lycopersicum*

According to occurrence records and environmental data, the current potentially suitable areas of two broomrapes are illustrated in Figure 2a,c. The simulated areas of potentially suitable for *O*. *cumana* were continuous and spanned the region from the eastern to western sectors of northern China (Figure 2a). Currently, high suitability areas were found to occur in north Xinjiang, central Inner Mongolia and Shaanxi, west Gansu and Jilin, and most of Ningxia province. The moderately suitable areas included south Heilongjiang, west Liaoning, and central Inner Mongolia. The areas with high and moderate fitness reached 318.69 × 10^4^ km^2^, occupying 33.08% of China's total area (Table 2). For *P*. *aegyptiaca*, the potentially suitable area is mainly distributed in Xinjiang. The areas were estimated to encompass ca. 30.64 × 10^4^ km^2^ (Figure 2c).

**TABLE 2:** The percentage of different classes of potentially suitable areas and risk zones of *O*. *cumana* and *P*. *aegyptiaca* under different climate scenarios

Portion of area (%)	*O. cumana*								*P. aegyptiaca*							
	Suitable area				Risk zones				Suitable area				Risk zones			
	Not suitable	Low suitable	Moderate suitable	High suitable	Not risk	Low risk	Moderate risk	High risk	Not suitable	Low suitable	Moderate suitable	High suitable	Not risk	Low risk	Moderate risk	High risk
current	37.40	29.52	21.94	11.14	51.17	27.68	17.22	3.32	91.89	4.93	2.22	0.96	94.20	4.46	1.08	0.26
SSP126‐2050	42.90	28.42	20.66	8.02	56.65	24.45	17.00	1.90	92.82	4.24	2.12	0.82	94.79	3.84	1.13	0.24
SSP245‐2050	45.44	29.32	18.87	6.37	56.12	27.01	15.62	1.25	91.16	4.72	2.76	1.36	93.92	4.29	1.49	0.30
SSP585‐2050	47.23	29.68	17.87	5.22	58.12	25.63	15.03	1.21	92.72	4.59	1.95	0.74	94.69	4.27	0.92	0.12
SSP126‐2090	45.25	30.68	18.31	5.76	56.29	27.08	15.39	1.24	92.64	4.48	2.07	0.81	94.56	4.10	1.08	0.27
SSP245‐2090	49.59	28.76	17.03	4.61	59.26	25.15	14.26	1.32	92.54	3.82	2.82	0.81	94.15	4.22	1.50	0.13
SSP585‐2090	58.43	31.82	8.26	1.48	65.35	31.11	3.36	0.18	93.57	4.31	1.63	0.50	93.98	4.54	1.20	0.28

The potentially suitable areas of *H*. *annuus* and *S*. *lycopersicon* based on current environmental factors are shown in Figure S4 and Table S3. For *H*. *annuus*, Maxent identified an area of 116.89 × 10^4^ km^2^ as highly suitable and 206.11 × 10^4^ km^2^ as moderately suitable, while 218.92 × 10^4^ km^2^ of the area was observed as low suitable. The potential distribution of *H*. *annuus* was concentrated in central and northern China, including the Shanxi, Ningxia, Shaanxi, Gansu, Inner Mongolia, Xinjiang, Jilin, Heilongjiang, and Liaoning. For *S*. *lycopersicum*, the projection results showed a wide range of potentially suitable areas in China. Of the total suitable area of *S*. *lycopersicum*, 9.31, 14.39, and 29.88% had high, moderate, and low suitability. The highly suitable areas for *S*. *lycopersicum* are predominantly distributed in Shandong, Hebei, Shanxi, Shaanxi, Henan, and Xinjiang.

According to the prediction results from MaxEnt, the potentially suitable areas of broomrape and its host plant were superimposed to obtain the risk zones of broomrape (Figure 2b,d). The results demonstrated that most of China's northern and central areas were risk areas for *O*. *cumana* (Figure 2b). Currently, the potential risk zones of *O*. *cumana* were predicted to occur in most of Shaanxi and Shanxi, central Inner Mongolia, northeast of Xinjiang, northern Jilin, Ningxia, and Gansu. For *O*. *cumana*, 20.54% of China's area, or approximately 197.88 × 10^4^ km^2^, is currently a potential middle‐ and high‐risk zone (Table 2). For *P*. *aegyptiaca*, the middle‐ and high‐risk zones were detected in Xinjiang (Figure 2d). The predicted area was around 12.91 × 10^4^ km^2^, 1.34% of China's area (Table 2).

### 3.4 Potential geographical distribution under future climate scenarios

Maxent predicted the future potentially suitable areas and risk zones of *O*. *cumana* and *P*. *aegyptiaca* under six climate change conditions (SSP126‐2050, SSP245‐2050, and SSP585‐2050; SSP126‐2090, SSP245‐2090, and SSP585‐2090) and then conducted the comparative analysis. The results were as follows:

1. *O. cumana*

**FIGURE 3:**
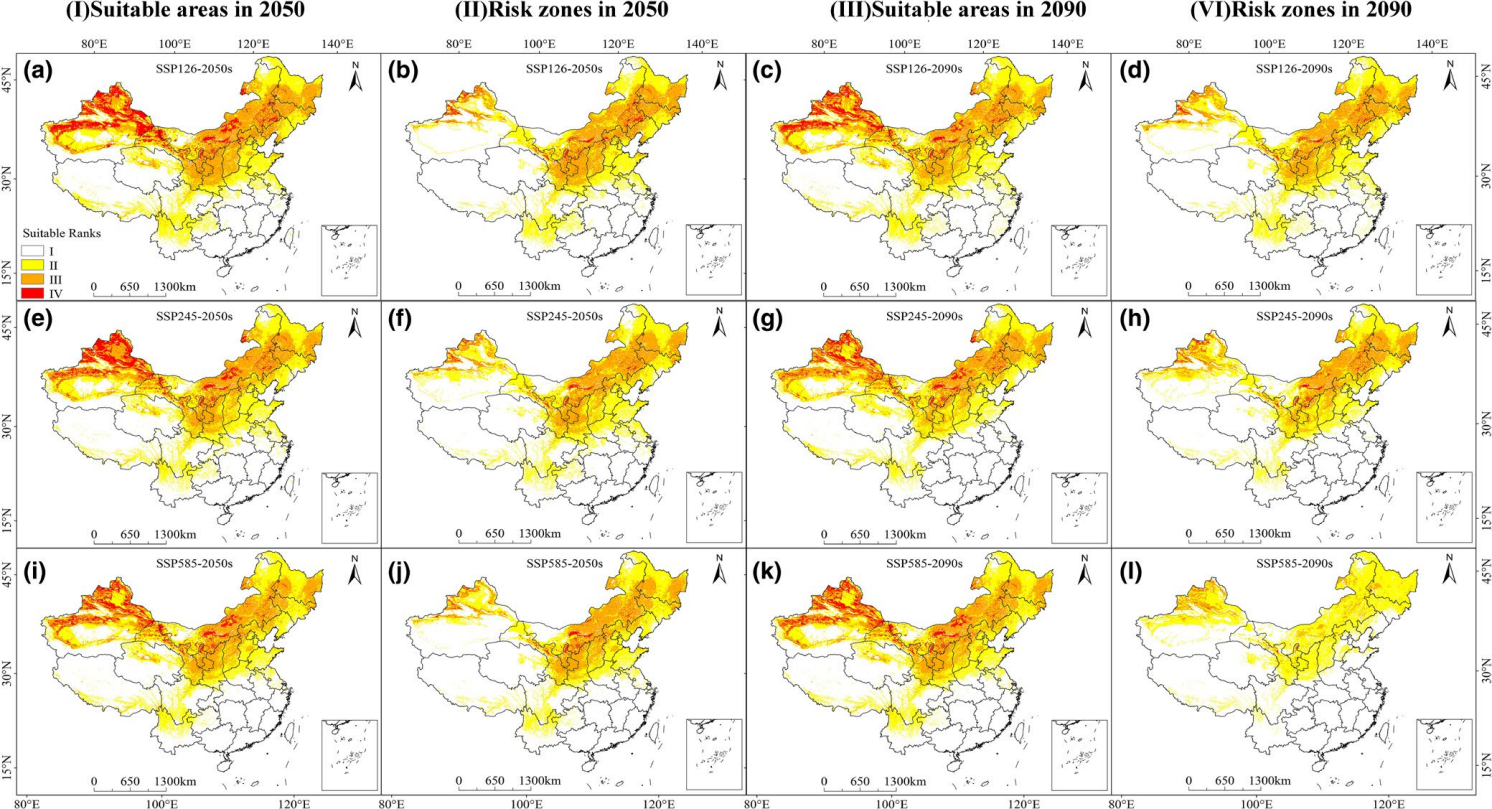
Future species distribution models (SDMs) of *Orobanche cumana* under climate change scenarios SSP126, SSP245, and SSP585. a, e, and i are maps of potentially suitable areas of *O*. *cumana* in 2050s; b, f, and j are maps of potentially risk zones of *O*. *cumana* in 2050s; c, g, and k are maps of potentially suitable areas of *O*. *cumana* in 2090s; d, h, and l are maps of potentially risk zones of *O*. *cumana* in 2090s

The calculated results for the *O*. *cumana* habitat suitability in SSP126, SSP245, and SSP585 are illustrated in Figure 3‐Ⅰ, Ⅲ, and Table 2. The results suggested that the future climate will adversely influence the predicted distribution of *O*. *cumana*. The potentially suitable areas of *O*. *cumana* declined in response to climatic warming (Figure S6‐a). Future highly suitable areas were mainly concentrated in Xinjiang and Inner Mongolia. In 2050, the area with habitat suitability accounted for 28.68% and 25.24% of the current predicted area under the SSP126 and SSP245 scenarios, respectively. The potential geographical distribution of *O*. *cumana* showed that suitable areas would decrease under scenario SSP585‐2050 to 222.45 × 10^4^ km^2^, covering about 23.09% of China's total area. Under the scenarios in 2090, the changing trend of suitable areas was consistent with 2050; the moderately and highly suitable areas decreased from 24.07% to 9.74%.

For the host plant of *O*. *cumana*, with proceeding climate change, there was no shift in the location of potential areas for *H*. *annuus*, but the highly suitable areas would significantly decrease for SSP585‐2090 (Figure S5‐I, Ⅱ and Table S3). In 2050, the reduction in highly suitable areas for *H*. *annuus* would be 15.09% and 5.39% under the SSP245 and SSP585 scenarios, respectively. In 2090, the high potential suitable areas of *H*. *annuus* would be decreased by 59.68% under SSP585, the predicted area to be around 47.13 × 10^4^ km^2^.

Similarly, the simulated results of *O*. *cumana* potential risk zones in different climate conditions showed that *O*. *cumana* risk zones decreased significantly with the changing SSPs. The warmer the climate, the lower the risk zones (Figure 3‐Ⅱ, Ⅳ; Table 2). In 2050 and 2090, the risk zones for *O*. *cumana* were widely located in the north of China, including Xinjiang, Inner Mongolia, and Shanxi. Under the SSP126‐2050, MaxEnt predicted that moderate‐ and high‐risk area was 182.08 × 10^4^ km^2^, with 18.90% of China's total area. The risk zones were expected to have a considerably contracted range in all regions, especially SSP585‐2090. The area of moderate and high risk was predicted to decrease significantly to 34.10 × 10^4^ km^2^, and 96.46% of the regions will be low or no risk regions.

1. *P. aegyptiaca*

**FIGURE 4:**
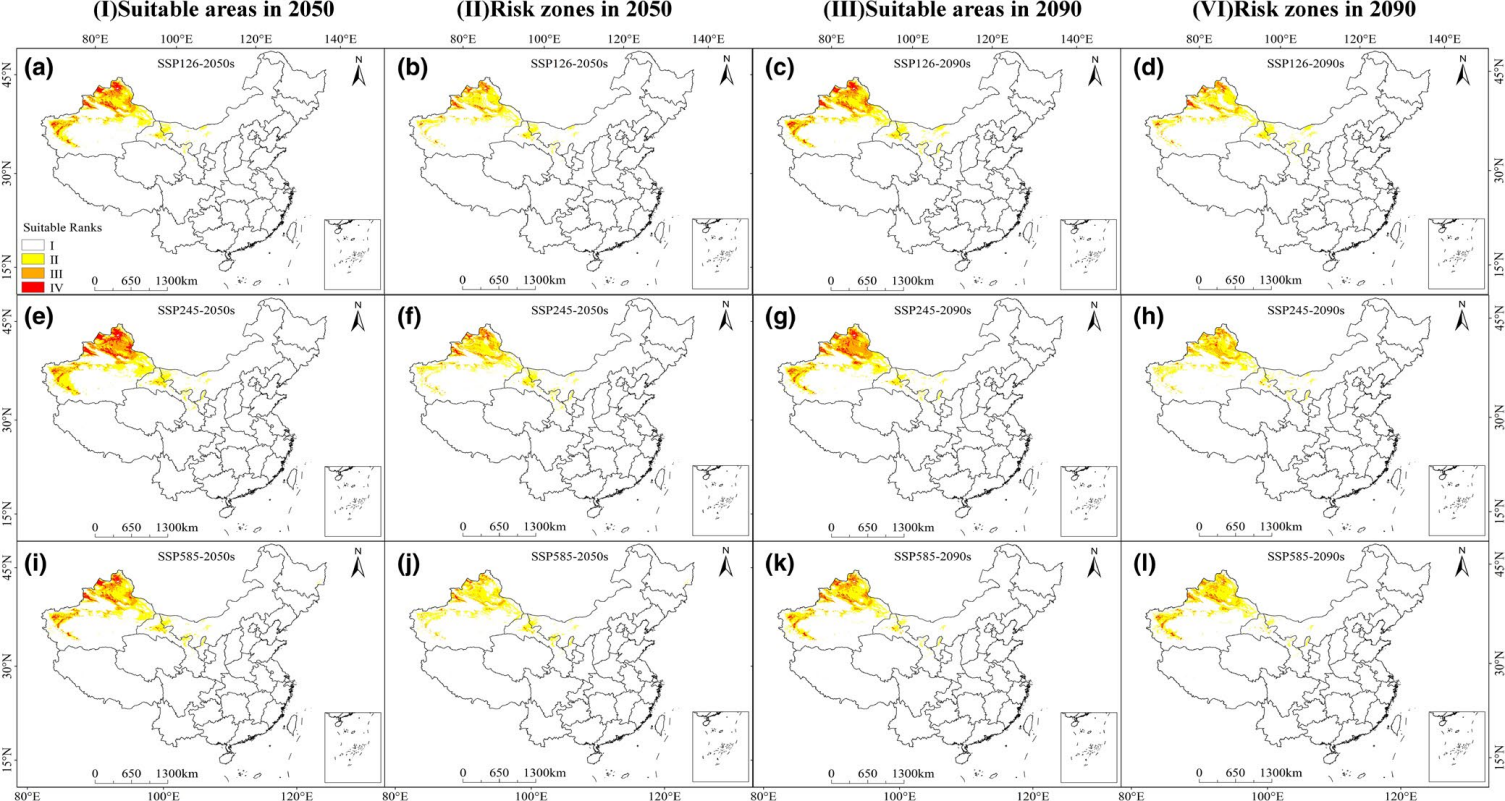
Future species distribution models (SDMs) of *Phelipanche aegyptiaca* under climate change scenarios SSP126, SSP245, and SSP585. a, e, and i are maps of potentially suitable areas of *P*. *aegyptiaca* in 2050s; b, f, and j are maps of potentially risk zones of *P*. *aegyptiaca* in 2050s; c, g, and k are maps of potentially suitable areas of *P*. *aegyptiaca* in 2090s; d, h, and l are maps of potentially risk zones of *P*. *aegyptiaca* in 2090s

The distribution maps of suitable areas for *P*. *aegyptiaca* in future climate conditions for 2050 and 2090 are summarized in Figure 4‐Ⅰ, Ⅲ, and Table 2. Compared with current climate conditions, the total suitable area of *P*. *aegyptiaca* in SSP126 and SSP585 was projected to decline slightly but increase slightly under the SSP245 scenario (Figure S6‐b). Highly suitable areas were still concentrated in northern Xinjiang, and the suitable areas ranged from 20.52 × 10^4^ km^2^–39.69 × 10^4^ km^2^, occupying 2.13%–4.12% of China's area.

The predicted results of future areas’ suitability under different SSPs are shown that the changing climatic condition alters the distribution of suitable future areas of *S*. *lycopersicum* (Figure S5‐Ⅲ, Ⅵ and Table S3). Compared to the current potential distribution, for 2050, the slight increase in area for high suitability was found under SSP126, and a slight decrease was found under SSP245 and SSP585. In 2090, the highly suitable areas of *S*. *lycopersicum* increased with increasing temperature. Under SSP585, the high suitability area increased significantly to 193.75 × 10^4^ km^2^.

Figure 4‐Ⅱ, Ⅳ reflects the influences of climate conditions on the potential future risk zones for *P*. *aegyptiaca*. In future climate conditions, high‐risk regions were scattered in northern Xinjiang, except for SSP585‐2050, the area of the middle‐ and high‐risk zones expanded slightly relative to current climate scenarios (Figure S6‐b). Under the SSP585‐2050 scenario, the area of the middle‐ and high‐risk habitat was the least at about 10.02 × 10^4^ km^2^. Under the SSP245‐2050 scenario, the area of the middle‐ and high‐risk habitat was the most at about 17.24 × 10^4^ km^2^. Overall, the risk area of *P*. *aegyptiaca* was relatively small, with the highest area accounting for 1.79% of China's area.

Overall, the results gained in this research showed that the potentially suitable areas and risk zones of *O*. *cumana* would decrease significantly, and those of *P*.*aegyptiaca* would fluctuate slightly under future climate change conditions. Furthermore, compared with *P*.*aegyptiaca*, *O*. *cumana* had a relatively more comprehensive distribution range; the middle and high suitability areas of *O*. *cumana* were obviously larger than those of *P*. *aegyptiaca*. Xinjiang and Inner Mongolia were the most severely affected place by *O*. *cumana* and *P*. *aegyptiaca*.

### 3.5 Future changes in risk zones

**FIGURE 5:**
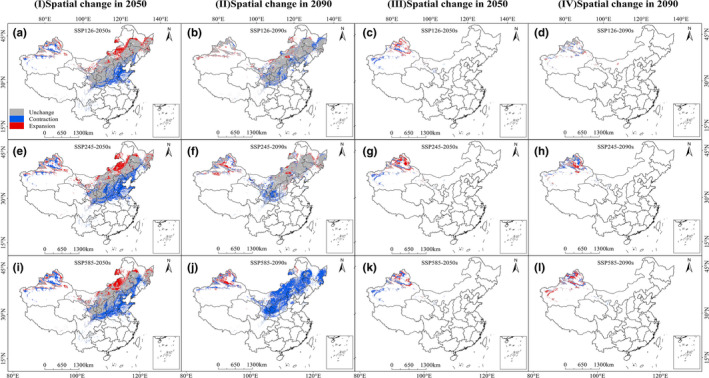
The spatial pattern changes of *Orobanche cumana* (a, e, i‐2050; b, f, j‐2090) and *Phelipanche aegyptiaca* (c, g, k‐2050, d, h, l‐2090) risk zones in different periods

Compared with the current risk zones, the predicted area of *O*. *cumana's* risk zones shrank significantly under six future combinations (Figure 5‐Ⅰ, Ⅱ, and Table S4). The parts of *O*. *cumana's* range with high risks of areas decrease were mainly concentrated in Hebei, Beijing, Liaoning, Shanxi, part of Shaanxi, and Xinjiang, and the contraction generally increased with decreasing latitude. Under the SSP585‐2090s condition, the loss of areas was the most severe at about 130.28 × 10^4^ km^2^, a loss of 65.83% of the current risk zones. The high‐risk areas under this condition were relatively small and the risk areas in Shanxi and Shaanxi were almost exhausted, as were the highly suitable areas in Inner Mongolia. This indicated that *O*. *cumana* risk zones would contract to a large degree, and the high‐risk range will only remain in Xinjiang under this scenario. In 2050, most predicted increases in risk zones were restricted to Inner Mongolia and Xinjiang, which amounted to ca. 34.79 × 10^4^ km^2^–40.73 × 10^4^ km^2^. Under the climate change conditions in 2090, the expansion areas were significantly smaller than in 2050 at 10.38 × 10^4^ km^2^–18.75 × 10^4^ km^2^. Overall, the total potential risk areas declined under the six future climatic scenarios, with the most pronounced contractions in Shanxi and Hebei.

Under the SSP126‐2050 and SSP585‐2050 scenarios, MaxEnt predicted that *P*. *aegyptiaca* total losses would occur mainly in western Xinjiang, with 0.07 × 10^4^ km^2^ and 3.36 × 10^4^ km^2^, respectively. Conversely, in SSP245‐2050, the total risk habitats expanded by 4.52 × 10^4^ km^2^, and the new areas were widely concentrated in northern Xinjiang, which indicated that a lot of the low‐risk areas under this condition might be replaced by the middle‐risk suitable areas (Figure 5‐Ⅲ and Table S4). By 2090, the MaxEnt model predicted total losses of 6.51% and 12.17% of the current area under the SSP126 and SSP245 scenarios, respectively. The distribution of newly added risk zones was also concentrated in northern Xinjiang under the SSP585‐2090s combination but was slightly less than the scenario of SSP245‐2050s at an increase of 4.37 × 10^4^ km^2^ (Figure 5‐Ⅳ and Table S4).

### 3.6 Changes in the distribution core of the risk zones

**FIGURE 6:**
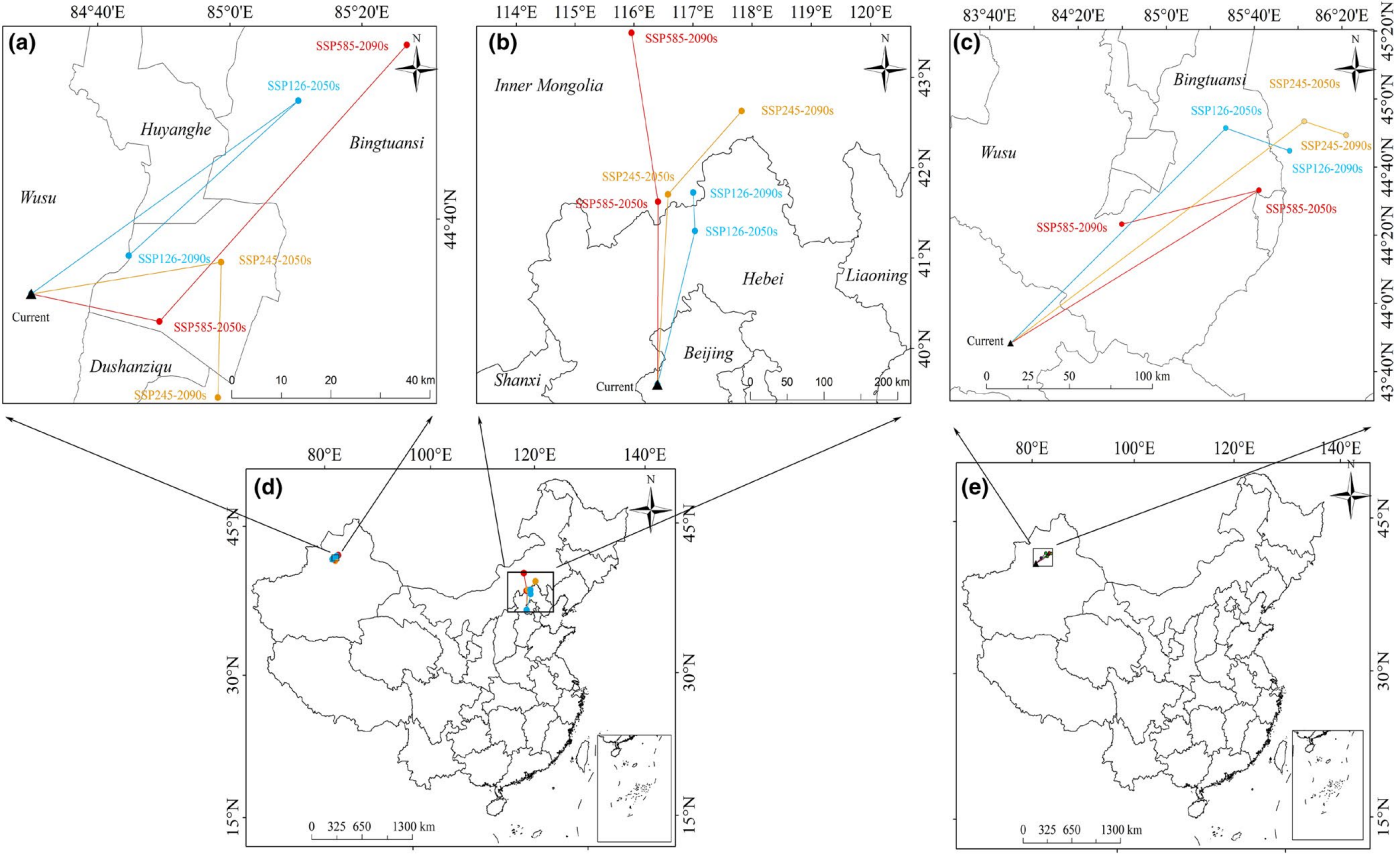
Predicted current distribution model (d, e) and the core distributional shifts (a, b, c) under different climate scenarios/years for *Orobanche cumana* (a, b, d) and *Phelipanche aegyptiaca* (c, e). The arrow indicates the magnitude and direction of predicted change over time

Currently, the distribution centroid of *O*. *cumana* was located in Xinjiang and Beijing province (Figure 6d). For the Xinjiang potential risk zones, the core of the current risk zone was located at 84°37′E and 44°23′N (Figure 6‐a; Table S5). The centroid of the risk zones shifted northeast to Bingtuansi (85°12′E, 44°48′N) under SSP126‐2050, but under SSP126‐2090, the centroid shifted to the southwest (84°51′E, 44°29′N), which was a migration distance of 44.86 km. Under SSP245, by the 2050s, the center of the risk zones moved northeast to Dushanziqu (85°5′E, 44°31′N), and by the 2070s, it shifted 25.25 km southward to 85°8′E and 44°17′N. At the same time, it was predicted that under SSP585, the centroid of the risk zones changed to 84°57′E and 44°23′N by 2050 and 85°27′E and 44°56′N by 2090. For the risk zones of Beijing, the core of the current risk zone was located at 115°47′E and 39°59′N (Figure 6‐b). Under all future climate scenarios (SSP126, SSP245, and SSP585), we saw that the centroid of the risk zones shifted toward the north overtime. Under the SSP585‐2090 scenario, the migration distance was the longest at about 439.28 km, and the center of the risk habitats moved north to Inner Mongolia (115°57′E, 43°56′N). Under the SSP126‐2050 scenario, the migration distance was the shortest at approximately 198.15 km, and the center of the risk zones shifted northeast to Hebei (116°39′E, 41°39′N). In short, under the future conditions, all the centroid of the potential risk zones were located at more northern areas with higher latitudes, except for the SSP245‐2090 Xinjiang risk zones.

For *P*. *aegyptiaca*, the current centroid of the potential risk zone was located in Xinjiang province, with geographical coordinates of 84°17′E and 43°25′N (Figure 6‐c, e; Table S5). The core of the future risk zones shifted to the position (85°35′E, 44°42′N) in the northeast under SSP126‐2050 and the position (86°6′E, 44°39′N) under SSP126‐2090. Under SSP245‐2050, the core of the future risk zones was located at a more northeast position (86°10′E, 44°49′N), and even more northeast at the position (86°30′E, 44°47′N) under SSP245‐2090. The potential risk zones center under SSP585‐2050 also shifted to northeast regions (85°55′E, 44°26′N) and high latitude. In contrast, the centroid under SSP585‐2090 shifted to southwest areas and a low latitude (84°56′E, 44°7′N). Overall, we found that the centroid of risk zones shift expressed a northeast tendency under the future six climatic scenarios, and the migration distance was relatively small, about 94.09 km under SSP585‐2090.

Evidently, in the future, the distribution centers of *P*. *aegyptiaca* and *O*. *cumana* are likely to shift to high latitudes.

## 4 DISCUSSION

Broomrapes have been listed as entry plant quarantine pests and agricultural plant quarantine pests in China (Wu & Qiang, 2006). The broomrapes in farmland significantly reduce agricultural productivity and inflict severe economic damage. Sunflower and tomato are the crops most seriously damaged by broomrapes in practical production. *Orobanche cumana* and *P*. *aegyptiaca* have become severe impediments to developing the local sunflower and tomato industries. Thence, the potential suitability maps of their extension are urgently needed for economic and quarantine purposes. This research carefully analyzed the suitable areas and risk zones for *O*. *cumana* and *P*. *aegyptiaca* under present and future climate changes. Our results provided a basis for decision‐making for the effective management and control of these notorious agricultural invasive weeds.

The predictive models constructed with MaxEnt were highly consistent with the known distribution of *O*. *cumuan* and *P*. *aegyptiaca* in China. *Orobanche cumana* was reported mainly on sunflower in Inner Mongolia, Xinjiang, Heilongjiang, Jilin, Shanxi, Shaanxi, Hebei, Gansu, and Ningxia provinces (Shi & Zhao, 2020; Wu et al., 2020). The model's simulation results highlighted that the suitable areas of *O*. *cumana* in the current climate condition were distributed in central Inner Mongolia, northern Xinjiang, northwestern Gansu Province, most of Ningxia province, and the potential area we predicted was broader. Meanwhile, our model showed that the moderately and highly suitable areas amount to 318.69 × 10^4^ km^2^ for *O*. *cumana* and the central location was in Xinjiang and Beijing. This result agrees with previous research that found that *O*. *cumuan* has a high potential for invasion in China (Mohamed et al., 2006). For *P*. *aegyptiaca*, Xinjiang is the area with the broadest distribution that suffers the greatest harm in China (Zhang et al., 2012). Similarly, our modeling demonstrated that the potentially suitable areas are currently concentrated in Xinjiang, with an area of 30.64 × 10^4^ km^2^. From the perspective of the overall ecological suitability of *O*. *cumuan* and *P*. *aegyptiaca* in China, the suitable areas of *P*. *aegyptiaca* in future climatic changes would decline slightly relative to the current habitat, except for under SSP245 climate condition. For *O*. *cumana*, the prediction results of habitat suitability in SSP126, SSP245, and SSP585 showed that the *O*. *cumana* suitable areas declined significantly as global warming intensity proceeded. This indicated a declining trend for *O*. *cumana* distribution in the future. Global warming seems to have limited the development of *O*. *cumana* and have a negative impact on the suitable areas for *O*. *cumana*. The decreases in the distribution of *O*. *cumana* may be due to the narrow range of optimum temperatures required for its conditioning and germination, which was found to be around 25–30°C: both lower and higher temperatures resulted in poor germination (Shi et al., 2018). Meanwhile, this also may be attributed to transitory or constantly high atmospheric temperatures causing a series of heat injuries, physiological disorders, and morphological and biochemical changes in plants (Lipiec et al., 2013).

Climate is a critical factor in predicting the potential distribution of species. However, the distribution of susceptible host plants is another important constraint for predicting and analyzing parasitic plants, particularly for holoparasitic plants. This study also predicted the potentially suitable areas in China of broomrapes’ susceptible host plants, using the MaxEnt. The predictions obtained for *H*. *annuus* and *S*. *lycopersicum* were concordant with the known distributions of these species (Fu et al., 2019). Throughout China, highly favorable conditions existed for both *H*. *annuus* and *S*. *lycopersicum*. Comparison of prediction results of broomrapes and their host plants under different climate scenarios showed that the projection suitable areas are similar in spatial extent. The suitable areas of *O*. *cumana* and host plant‐*H*. *annuus* were all distributed in central and northern China, including the Shanxi, Ningxia, Shaanxi, Gansu, Inner Mongolia, Xinjiang, Jilin, Heilongjiang, and Liaoning. For *P*. *aegyptiaca and S*. *lycopersicum*, Xinjiang is the common suitable area of the two species. There is a higher risk of the invasion of broomrapes in the co‐occurrence areas, which may affect agricultural development or require costlier control methods.

Our study overlapped the potentially suitable areas between broomrapes and selected susceptible host plants to analyze the risk zones of broomrapes. The risk zones for the future distribution of these two weeds indicated that they were influenced by extreme temperatures or precipitation and these areas are expected to either increase or decrease as the climate changes. It was observed that, for *P*. *aegyptiaca*, the risk zones would rise slightly under varying future climate conditions, which indicated that extreme scenarios had little effect on *P*. *aegyptiaca*. However, for *O*. *cumana*, our model suggested that moderate and high‐risk zones in all six future climatic conditions would decline significantly relative to the current risk zones. The warmer the climate was, the lower the risk zones. Meanwhile, our supplementary data showed that the highly suitable area of sunflower decreased with the increase of temperature, indicating that temperature had a negative effect on the suitable areas of the sunflower and consequently decreased the suitable area of *O*. *cumana* by limiting host development (Table S3). In the previous articles, some researchers also reported that the yield of sunflower (the host of *O*. *cumana*) significantly decreased under global warming (Johkan et al., 2011). This might reduce the risk zones of *O*. *cumana*. In addition, previous studies suggested that temperature impacts on the host–parasite relationship are complex (Sukno et al., 2001). Sukno et al. studied the effects of temperature on the resistance of sunflower to parasitism by *O*. *cumana* and found that the level of parasitism was lowest for all the treatments at the highest temperature (Sukno et al., 2001). These results have certain similarities with those of Eizenberg et al. (2003) and Eizenberg et al. (2003), who found that under controlled conditions in polyethylene bags and Petri dishes, as the temperature rose, more *Orobanche* tubercles degenerated and died; Namely, the sunflower plants expressed higher levels of resistance. The defense mechanism may have increased with rising temperature, causing the broomrapes to develop retardation, necrosis, or die. Taken together, the suitability of conditions for the host can also influence the future suitability of conditions for parasites.

However, incorporating biotic interactions (predation, competition, parasitism, and mutualism) in SDMs does not always improve predictive accuracy (Pellissier et al., 2010; Silva et al., 2014), as it requires extensive knowledge regarding biotic interactions and population‐level impacts, as well as detailed and specific information in biotic interactors and target species (Fordham et al., 2012; Simões & Peterson, 2018). In this study, despite the broomrapes and selected host plants have been thorough a detailed prediction of their potential distribution, the essence of their interaction remains poorly known. Additionally, different/novel host plants may influence an invasive species range. *Helianthus annuus* and *S*. *lycopersicum* are not the only wild host plant of *O*. *cumana* and *S*. *lycopersicum*, respectively. Broomrapes can parasitize many hosts, such as tobacco and muskmelon (Parker, 2012). We suggest that identifying the broomrapes–host interaction and predicting the potential distributions of additional wild hosts would aid future assessment of risks of broomrapes invasion.

The precise environmental biology of broomrapes and their phenological synchrony with host plants still require more investigation. Even so, the impacts of climate limits and climate change on broomrape distribution and the host–parasite relationship are unequivocal. Temperature, precipitation, and soil properties are three bioclimatic features that can be useful starting points to study the mechanisms with which the climate affects species distribution (Hu et al., 2015). During the broomrapes’ life cycle, the temperature is the dominant factor affecting their growth, and there is a strong interdependence between parasitic ability and temperature (Eizenberg & Goldwasser, 2018). Ephrath and Eizenberg (2010) reported that the relationship between the process of *O*. *cumana* and *P*. *aegyptiaca* attachment and development and thermal time followed a sigmoid‐shaped curve, and the broomrapes’ biomass was also found to be closely correlated to thermal time. Research on the influences of temperature on the germination of different species of broomrapes showed that each broomrape had a specific best temperature range for germination and growth, which generally reflected its geographical distribution (Song et al., 2005). The optimal temperatures for both conditioning and germination were about 25–30°C for *O*. *cumana* and about 18–21°C for *P*. *aegyptiaca* (Shi et al., 2018; Song et al., 2010). Final germination must fulfill the following two conditions: the minimum average temperature that must be exceeded and the maximum temperature above which the seeds will not germinate (Ermias & Murdoch, 1999). Therefore, appropriate adjustment of the sowing date can considerably reduce the abundance of broomrapes and lessen the damage caused by broomrapes to host plants.

Humidity is another major factor affecting broomrapes seed germination. The germination of broomrapes seeds requires pre‐culturing under specific temperature and humidity conditions (Kebreab & Murdoch, 1999). When the soil humidity is too high, the parasitism and growth of broomrapes are inhibited, which may be because the humidity affects the dormancy characteristics of broomrapes, and then affects their germination and parasitism (Kebreab & Murdoch, 2000). Less infestation due to broomrapes has been observed in sunflower/tomato grown under flooded irrigation or in years with more precipitation (Punia, 2014). In addition, a substantial reduction in seed viability/longevity was observed when the seeds were imbibed and at higher temperatures. Thus, combining irrigation with solarization during the dry season could significantly raise the depletion of the soil seed bank.

Soil conditions also have a significant influence on shaping the distribution range of broomrapes. Alkaline soil (pH > 7.0) was found to be favorable for broomrape survival and growth, but the parasitism rate was less in acidic soil (pH < 7.0) (Dinesha & Dhanapal, 2013; Di et al., 2020). Shi et al. (2018) reported that sandy loamy soil with a temperature of around 25–30°C, moisture of 60%–70%, and soil pH value of 8 could benefit the attachment and growth broomrapes. Some research results have revealed that the parasitism rates of broomrapes were closely related to the amount of available soil nitrogen and the type of nitrogen fertilizer used (Irmaileh, 1994; Mesbah et al., 2012). As soil nitrogen rates increased, the numbers and dry weights of broomrape shoots declined, the yields of sunflower/tomato substantially rose, and urea fertilizers could significantly reduce the occurrence of broomrapes (Mariam & Suvvanketnikom, 2004; Narges & Syyed, 2014). Therefore, N should be applied at least at the recommended rate if using N at higher doses is not possible on the ground of affordability by farmers and cost economics. In this study, the CMIP6 climatic scenarios were employed to determine the potential effects of climate change on the environmental suitability for broomrapes. Among the 19 environmental variables used for the developed model, temperature, precipitation, and soil properties all made important contributions to the habitat suitability of *O*. *cumana* and *P*. *aegyptiaca*, suggesting that these factors play essential roles in broomrapes distribution.

Responses to climate change in biological communities are usually related to species distribution about latitude or extreme elevations (Lenoir et al., 2008). Generally, with the climate changes, suitable areas in the Northern Hemisphere are expected to expand northward, and these areas will become more climatically suitable (Wang et al., 2017). Some studies have revealed that some species may expand their ranges to higher latitudes and elevations as global warming continues (Bertrand et al., 2011; Walther et al., 2005; Wilson et al., 2007). The study suggested that a northward shift of broomrapes’ risk zones to higher latitudes would gradually become more significant. The centroid of risk zones for *O*. *cumuan* and *P*. *aegyptiaca* shifted to areas at higher latitudes than their current habitat, which may be facilitated through adaptations. Therefore, the long‐term risks are greater in higher latitudes, as these areas will experience warming, which will promote the invasion and establishment of *O*. *cumuan* and *P*. *aegyptiaca*.

## 5 CONCLUSIONS

In this research, we established a predictive model that considered climate, soil, and topography and provided a basis for assessing the suitable area of *Orobanche cumana* and *Phelipanche aegyptiaca*. This model was applied to examine the influences of climate change on the potentially suitable area for those two broomrapes in China. We also took global climate change and host plants into account to examine the potential risk status of the broomrapes in the future. The results showed that Xinjiang and Inner Mongolia would experience the greatest risk of establishing the *O*. *cumana* and *P*. *aegyptiaca*, which may affect local agricultural productivity or pay a greater price to control these weeds. Although the results suggested that the distribution of risk zones for *O*. *cumuan* will be significantly decreased with global warming, prevention and treatment should not be taken lightly due to the rapid spread of broomrapes and the difficulty of eradication. The predicted results can be applied to clearly understand the distribution of suitable areas and risk zones of two broomrapes and determine their expansion reasons to provide a useful reference in formulating quarantine and control measures for *O*. *cumana* and *P*. *aegyptiaca*. Future studies could be needed to consider the effects of human behavior or the habitats of other host plants, and even the study area could be extended into the global scope.

## AUTHOR CONTRIBUTIONS

**Lu Zhang:** Writing – original draft (equal); Writing – review & editing (equal). **Sifeng Zhao:** Project administration (lead); Supervision (lead). **Xiaolei Cao:** Formal analysis (equal). **Zhaoqun Yao:** Conceptualization (equal). **Xue Dong:** Investigation (equal). **Meixiu Chen:** Data curation (equal). **Lifeng Xiao:** Software (equal).

## Supporting information

Supplementary Material

## Data Availability

Climate data, MaxEnt input files and the four species’ distribution data: Dryad https://doi.org/10.5061/dryad.8pk0p2nph; and the data that supports the findings of this study are available in the Appendix S1 of this article.
