# Supplementary material for: Identification of risk areas for Orobanche cumana and Phelipanche aegyptiaca in China, based on the major host plant and CMIP6 climate scenarios
Source: Ecol Evol. 2022 Apr 19;12(4):e8824. doi: 10.1002/ece3.8824 (PMC9018459; doi:10.1002/ece3.8824)
Supplement: Supplementary file 1 — Supplementary Material [file ECE3-12-e8824-s001.docx]

**Supplementary Information**

**The scientific publications of species’ occurrence data**

Committee of Fora of China, Chinese Academy of Sciences (1990) Flora of China Volume 69. Beijing: Science Press, 97-124.

Cui HX, Wang N, Long XQ, An K, Hou M, Cui WD (2020) Review of the species, hazard and management status of *Orobanche* L. in Xinjiang. Plant Quarantine, 34(03):20-24. https://doi.org/10.19662/j.cnki.issn1005-2755.2020.03.004

Editorial Committee of Flora of Xinjiang (1993) Flora of Xinjiang. [Xinjiang Sci-Tech and Public Health Press](http://dict.cn/Xinjiang%20Sci-Tech%20and%20Public%20Health%20Press), 429-438.

Fu MQ, Liu B, Wang J, Sun Y, Wang XH, Cheng F (2019) Temporal and spatial changes of sunflower production in China from 1985 to 2015. Journal of Henan Agricultural University, 53(04):630-637. https://doi.org/10.16445/j.cnki.1000-2340.2019.04.019

Shi BX, Zhao J (2020) Recent progress on sunflower broomrape research in China. OCL, 27:30. https://doi.org/10.1051/ocl/2020023

The ministry of agriculture of the People's Republic of China (2021) Circular of the General Office of the Ministry of Agriculture on printing and distributing the Categorized List of Pests Distribution at County-level Region in China by Disease and Pest. Gazette of the Ministry of Agriculture and Affairs of the People’s Republic of China, 05:89-89.

The Rural Social and Economic Investigation Department of National Bureau of Statistics (2020) China Rural Statistical Yearbook. China Statistics Press.

Wang YJ, Ji LJ, Li QS, Wang LS, Pan JH, Kong LX (2015) Investigation and identification of parasitic weed Orobanche L. Weed Science, 33(03):6-10. https://doi.org/10.19588/j.issn.1003-935x.2015.03.002

Wu WL, Jiang CL, Huang ZF, Zhang CX, Bai QJ, Yun XP, Wei SH, Huang HJ (2020) Investigation of present occurrence and infestation of *Orobanche cumana* Wallr. in China. Plant Protection, 46(3):266-273. https://doi.org/CNKI:SUN:ZWBH.0.2020-03-042

Yao ZQ, Cao XL, Fu C, Zhao SF (2017) Review of the distribution and management technology of various *Orobanche* L. species in Xinjiang, China. Journal of Biosafety, 26(1), 23-29. <https://doi.org/10.3969/j.Issn.2095-1787.2017.01.004>

Yin ZQ, Zhou GL (1993) A higher Parasitic Plant in Xinjiang (Ⅱ) & Orabanchaceae. Journal of Xinjiang Agricultural University, 16(1):48-54.

Yun XP, Su YJ, Du L, Tian XY, Gao HY, Zhang G, Han L, Bai QJ (2021) Distribution and composition of physiological races of sunflower broomrape (*Orobanche cumana* Wallr.) in China. Weed Science, 39(1): 12-20. https://doi.org/10.19588/j.issn.1003-935X.2021.01.002

Zhang JL, Jiang Q (1994) The host and distribution of *Cuscuta* L. and *Orobanche* L. Plant Quarantine, 1994,8(2):69-73. https://doi.org/CNKI:SUN:ZWJY.0.1994-02-001

Zhang XK, Yao ZQ, Zhao SF, Ding LL, Du J (2012) Distribution, harmfulness and its assessment of *Orobanche aegyptiaca* in Xinjiang province. Plant Quarantine, 06:31-39. <https://doi.org/CNKI:SUN:ZWJY.0.2012-06-009>

Table S.1 List of environmental variables included in the Maxent algorithm for broomrapes and host plants.

| Type | Variables | Description | Units |
| --- | --- | --- | --- |
| Bioclimatic Variables | Bio1 | Annual Mean Temperature | ℃ |
|  | Bio2 | Mean Diurnal Range | ℃ |
|  | Bio3 | Isothermality | 1 |
|  | Bio4 | Temperature Seasonality | 1 |
|  | Bio5 | Max Temperature | ℃ |
|  | Bio6 | Min Temperature of Coldest Month | ℃ |
|  | Bio7 | Temperature Annual Range | ℃ |
|  | Bio8 | Mean Temperature of Wettest Quarter | ℃ |
|  | Bio9 | Mean Temperature of Driest Quarter | ℃ |
|  | Bio10 | Mean Temperature of Warmest Quarter | ℃ |
|  | Bio11 | Mean Temperature of Coldest Quarter | ℃ |
|  | Bio12 | Annual Precipitation | mm |
|  | Bio13 | Precipitation of Wettest Month | mm |
|  | Bio14 | Precipitation of Driest Month | mm |
|  | Bio15 | Precipitation Seasonality | 1 |
|  | Bio16 | Precipitation of Wettest Quarter | mm |
|  | Bio17 | Precipitation of Driest Quarter | mm |
|  | Bio18 | Precipitation of Warmest Quarter | mm |
|  | Bio19 | Precipitation of Coldest Quarter | mm |
| Top Soil Variable | T_GRAVEL | Topsoil Gravel Content | %vol. |
|  | T_SAND | Topsoil Sand Fraction | % wt. |
|  | T_SILT | Topsoil Silt Fraction | % wt. |
|  | T_CLAY | Topsoil Clay Fraction | % wt. |
|  | T_USDA_TEX_CLASS | Topsoil USDA Texture Classification | name |
|  | T_REF_BULK_DENSITY | Topsoil Reference Bulk Density | kg/dm^3^ |
|  | T_OC | Topsoil Organic Carbon | % weight |
|  | T_PH_H_2_O | Topsoil pH (H_2_O) | -log(H^+^) |
|  | T_CEC_CLAY | Topsoil CEC (clay) | cmol/kg |
|  | T_CEC_SOIL | Topsoil CEC (soil) | cmol/kg |
|  | T_BS | Topsoil Base Saturation | % |
|  | T_TEB | Topsoil TEB | cmol/kg |
|  | T_ESP | Topsoil Sodicity (ESP) | % |
|  | T_ECE | Topsoil Salinity (Elco) | dS/m |
| Topography | ELEV | Elevation | m |

Table S.2 Percentage contributions and permutation importance of the environmental variables included in the MaxEnt models for *H. annuus* and *S. lycopersicum*

| Variable | Description | *H. annuus* | | *S. lycopersicum* | |  |
| --- | --- | --- | --- | --- | --- | --- |
|  |  | Percent contribution | Permutation importance | Percent contribution | Permutation importance |  |
| Bio1 | Annual Mean Temperature | 20.0 | 33.4 | 37.2 | 47.4 |  |
| Bio12 | Annual Precipitation | 20.0 | 13.8 | 1.5 | 11.8 |  |
| ELEV | Elevation | 14.9 | 17.1 | 16.5 | 10.6 |  |
| Bio10 | Mean Temperature of Warmest Quarter | 9.7 | 4.0 | 9.7 | 1.9 |  |
| Bio19 | Precipitation of Coldest Quarter | 9.3 | 5.9 | ╳ | ╳ |  |
| T_TEB | Topsoil TEB | 5.9 | 1.9 | ╳ | ╳ |  |
| Bio5 | Max Temperature | 4.8 | 6.5 | 4.6 | 13.3 |  |
| T_SILT | Topsoil Silt Fraction | 4.8 | 3.2 | ╳ | ╳ |  |
| Bio6 | Min Temperature of Coldest Month | 4.4 | 7.4 | 5.1 | 7.0 |  |
| T_PH_H_2_O | Topsoil pH (H_2_O) | 3.0 | 0.3 | ╳ | ╳ |  |
| Bio11 | Mean Temperature of Coldest Quarter | 1.2 | 4.5 | ╳ | ╳ |  |
| T_BS | Topsoil Base Saturation | ╳ | ╳ | 3.6 | 3.5 |  |
| Bio2 | Mean Diurnal Range | ╳ | ╳ | 9.2 | 6.9 |  |
| T_SILT | Topsoil Silt Fraction | ╳ | ╳ | 5.0 | 1.3 |  |
| Bio5 | Max Temperature | ╳ | ╳ | 4.6 | 13.3 |  |
| T_BS | Topsoil Base Saturation | ╳ | ╳ | 3.6 | 3.5 |  |
| T_CEC_SOIL | Topsoil CEC (soil) | ╳ | ╳ | 3.1 | 1.2 |  |
| Bio15 | Precipitation Seasonality | ╳ | ╳ | 2.8 | 2.0 |  |
| T_OC | Topsoil Organic Carbon | ╳ | ╳ | 1.8 | 3.1 |  |
| T_ECE | Topsoil Salinity (Elco) | ╳ | ╳ | 1.5 | 1.7 |  |

Table S.3 The potential distribution areas of *H. annuus* (*O. cumana* host plant) and *S. lycopersicum* (*P.* *aegyptiaca* host plant) under different climate scenarios.

| Area  (×10^4^ km^2^) | Not  suitable | Low  suitable | Moderate suitable | High  suitable | Not  suitable | Low  suitable | Moderate suitable | High  suitable |
| --- | --- | --- | --- | --- | --- | --- | --- | --- |
| current | 421.49 | 218.92 | 206.11 | 116.89 | 447.20 | 287.85 | 138.68 | 89.68 |
| SSP126-2050 | 454.44 | 198.41 | 189.68 | 120.87 | 390.59 | 288.82 | 188.42 | 95.58 |
| SSP245-2050 | 406.97 | 208.88 | 248.31 | 99.24 | 384.74 | 293.36 | 198.78 | 86.53 |
| SSP585-2050 | 432.86 | 201.25 | 218.71 | 110.59 | 350.07 | 309.81 | 220.17 | 83.36 |
| SSP126-2090 | 420.90 | 192.92 | 245.07 | 104.52 | 387.00 | 293.09 | 189.21 | 94.11 |
| SSP245-2090 | 371.56 | 221.43 | 258.97 | 111.45 | 322.97 | 298.37 | 231.44 | 110.64 |
| SSP585-2090 | 385.55 | 314.39 | 216.34 | 47.13 | 187.77 | 319.40 | 262.47 | 193.75 |

Table S.4 Dynamics of changes in risk zones for *O. cumana* and *P. aegyptiaca* under six future climate scenarios/years.

| Area(×10^4^ km^2^) | *O. cumana* | | | | | *P. aegyptiaca* | | | |
| --- | --- | --- | --- | --- | --- | --- | --- | --- | --- |
|  | Expansion | Contraction | Unchanged | Total | Expansion | | Contraction | Unchanged | Total |
| SSP126-2050 | 34.79 | 49.58 | 141.83 | -14.79 | 6.61 | | 6.68 | 9.03 | -0.07 |
| SSP245-2050 | 40.73 | 74.93 | 116.23 | -34.20 | 11.15 | | 6.62 | 9.06 | 4.52 |
| SSP585-2050 | 39.62 | 78.89 | 111.74 | -39.27 | 6.13 | | 9.50 | 6.12 | -3.36 |
| SSP126-2090 | 11.46 | 33.84 | 143.46 | -22.38 | 2.25 | | 3.10 | 12.71 | -0.84 |
| SSP245-2090 | 18.75 | 30.82 | 125.27 | -12.07 | 6.58 | | 8.15 | 12.12 | -1.57 |
| SSP585-2090 | 10.38 | 130.28 | 21.06 | -119.90 | 9.86 | | 5.49 | 6.84 | 4.37 |

Table S.5 The centroid of *O. cumana* and *P. aegyptiaca* under different climate scenarios. Distance (the migration distance between the center points of the current and the future suitable hazard)

|  | *O.cumana* (Northwest) | | | *O.cumana* (Northeast) | | | *P. aegyptiaca* | | |
| --- | --- | --- | --- | --- | --- | --- | --- | --- | --- |
|  | longitude | latitude | distance | longitude | latitude | distance | longitude | latitude | distance |
| current | 84.62946 | 44.39840 | - | 115.78414 | 39.99363 | - | 84.28530 | 43.42677 | - |
| SSP126-2050 | 85.20810 | 44.81272 | 65.04 | 116.65601 | 41.65053 | 198.15 | 85.58775 | 44.70709 | 176.42 |
| SSP245-2050 | 85.09036 | 44.51972 | 39.08 | 116.27897 | 42.09390 | 236.93 | 86.17138 | 44.82116 | 216.31 |
| SSP585-2050 | 84.96415 | 44.39986 | 26.66 | 116.09964 | 42.02690 | 227.36 | 85.92283 | 44.43845 | 172.98 |
| SSP126-2090 | 84.85568 | 44.49702 | 21.08 | 116.69785 | 42.08043 | 244.15 | 86.10050 | 44.66228 | 200.02 |
| SSP245-2090 | 85.14646 | 44.29604 | 42.76 | 117.65713 | 42.91725 | 360.42 | 86.50615 | 44.79591 | 233.98 |
| SSP585-2090 | 85.45550 | 44.94308 | 89.19 | 115.95090 | 43.94653 | 439.28 | 84.94120 | 44.12774 | 94.10 |
| Average distance | - | - | 47.30 | - | - | 284.38 | - | - | 182.30 |


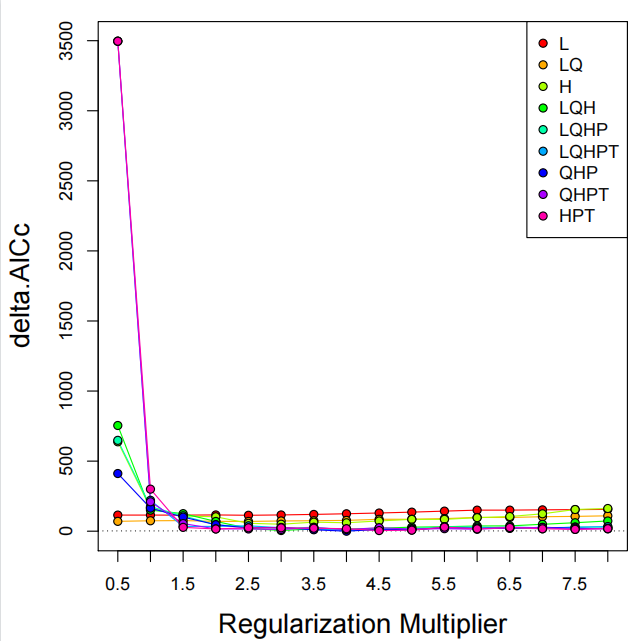


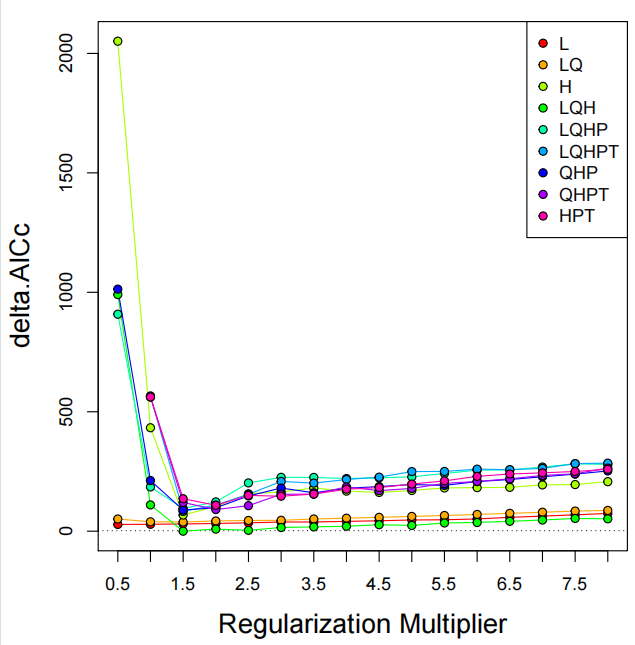


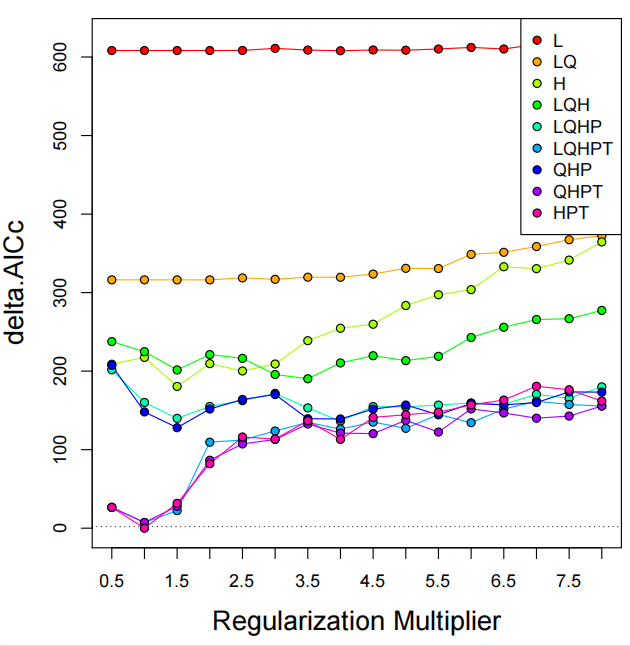


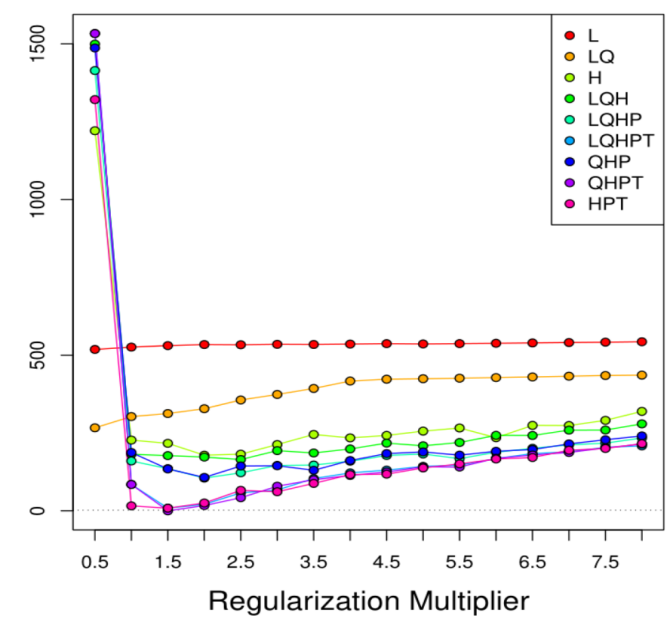


Fig. S.1. Performances of MaxEnt model of broomrapes and host plants under different settings. Red arrow indicates the delta AICc-chosen setting. Note: Red arrow indicates the delta AICc-chosen setting. L= Linear; Q= Quadratic; H= Hinge; P= Product; T= Threshold.

(a) *O. cumana*; (b) *P. aegyptiaca*; (c) *H. annuus*; (d) *S. lycopersicum*


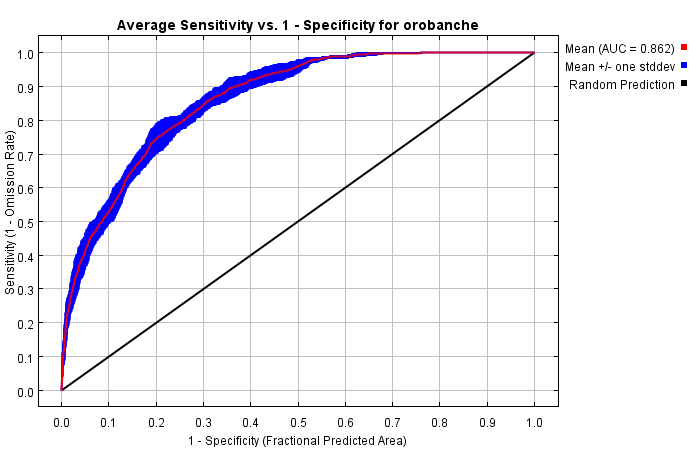


**(a)**


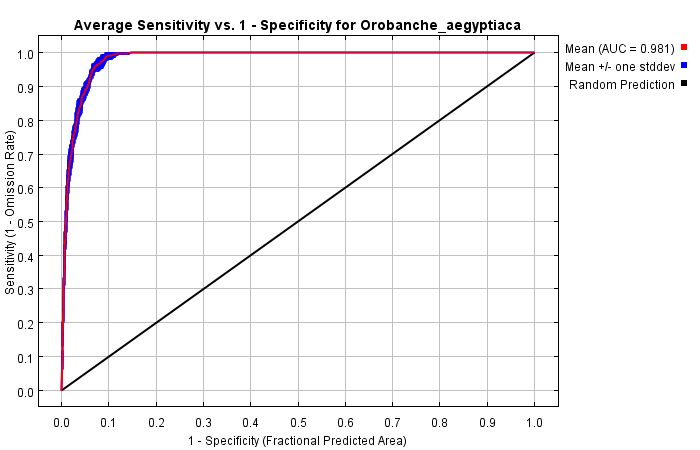


**(b)**


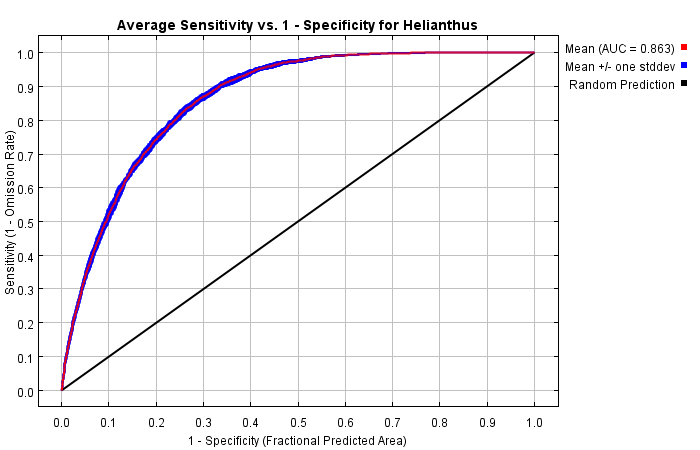


**(c)**


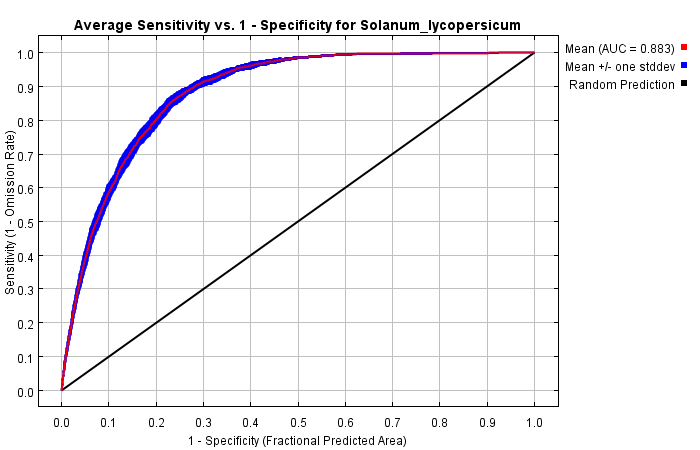


**(d)**

Fig. S.2. The ROC curve for broomrapes and host plants under current climate. (a) *O. cumana*; (b) *P. aegyptiaca*; (c) *H. annuus*; (d) *S. lycopersicum*


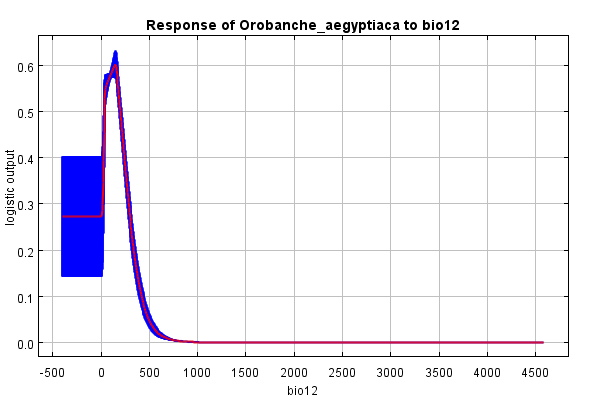

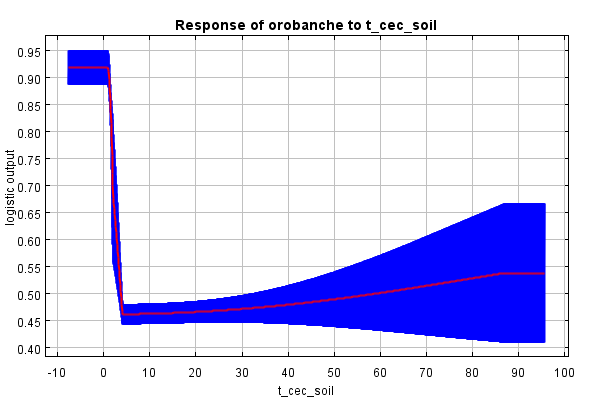

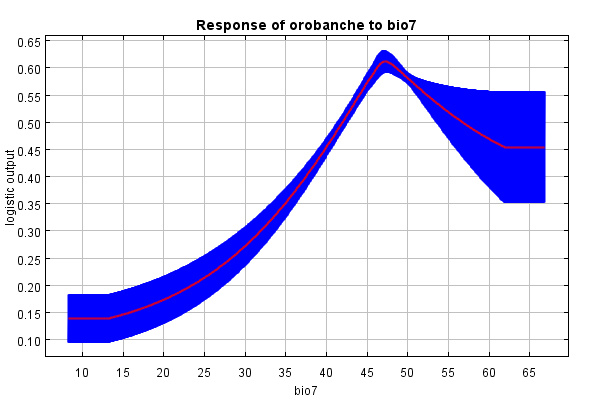

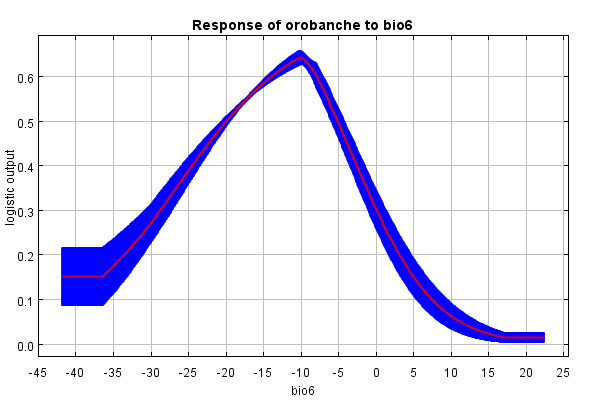

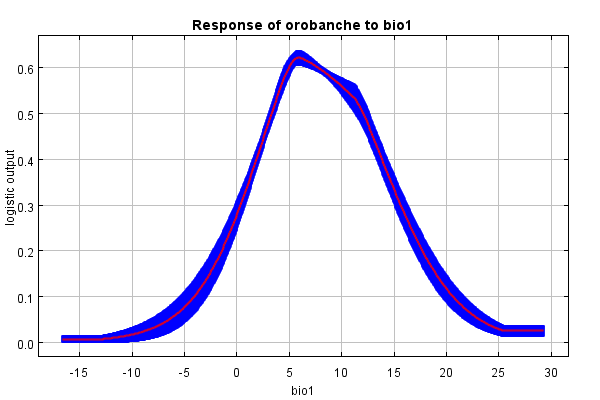

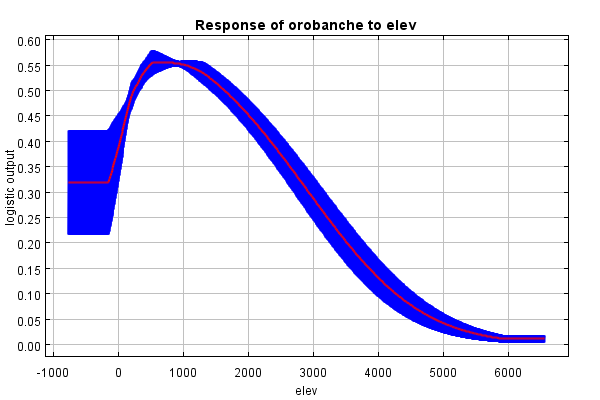

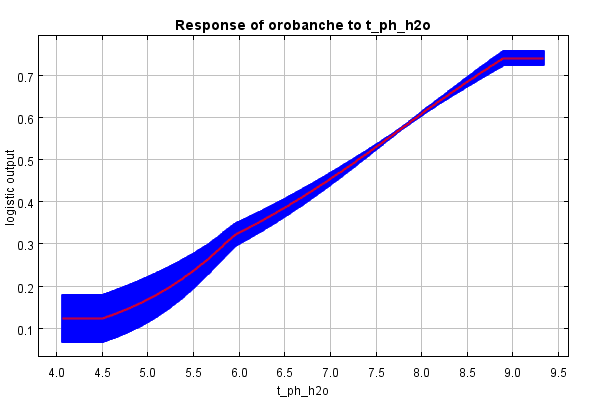


**(a)**

**(b)**

**(c)**


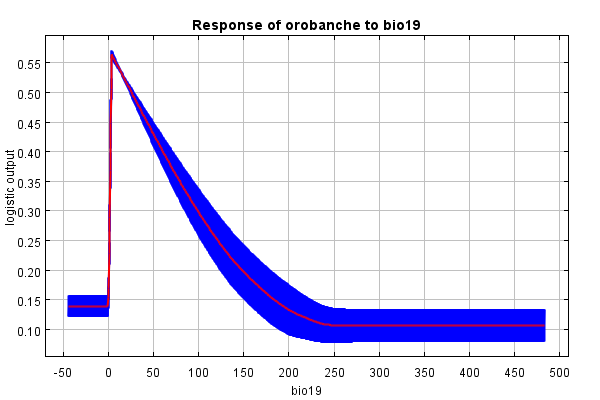


**(d)**

**(e)**

**(f)**

**(g)**

Bio1

Bio7

Bio19

ELEV

T_CEC_SOIL

T_PH_H_2_O


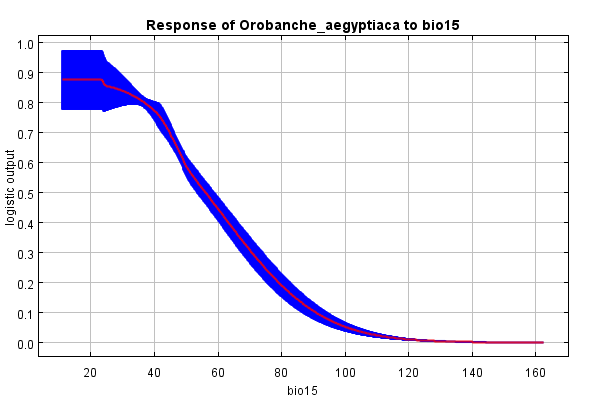

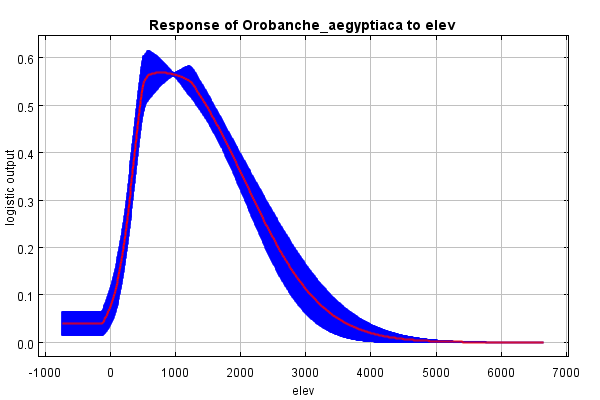

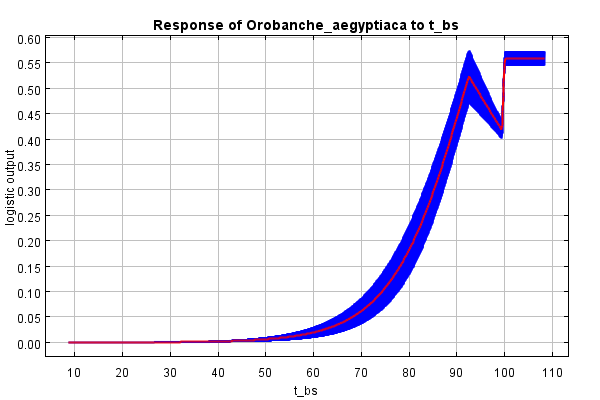

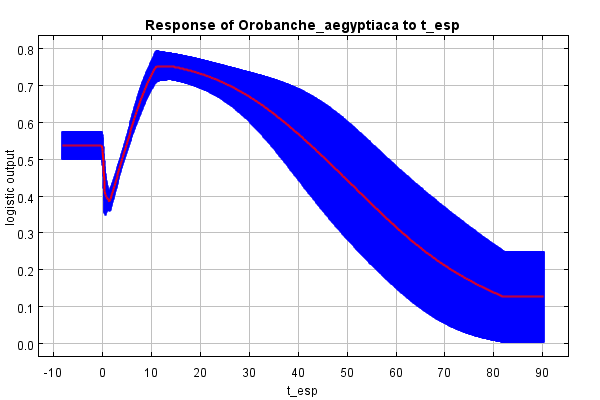


ELEV

Bio12

Bio15

T_BS

T_ESP

**(h)**

**(i)**

**(j)**

**(k)**

**(l)**

Bio6

Fig. S.3. Response curves for important environmental predictors in the species distribution model for *O. cumana* (a–g) and *P. aegyptiaca* (h–l).


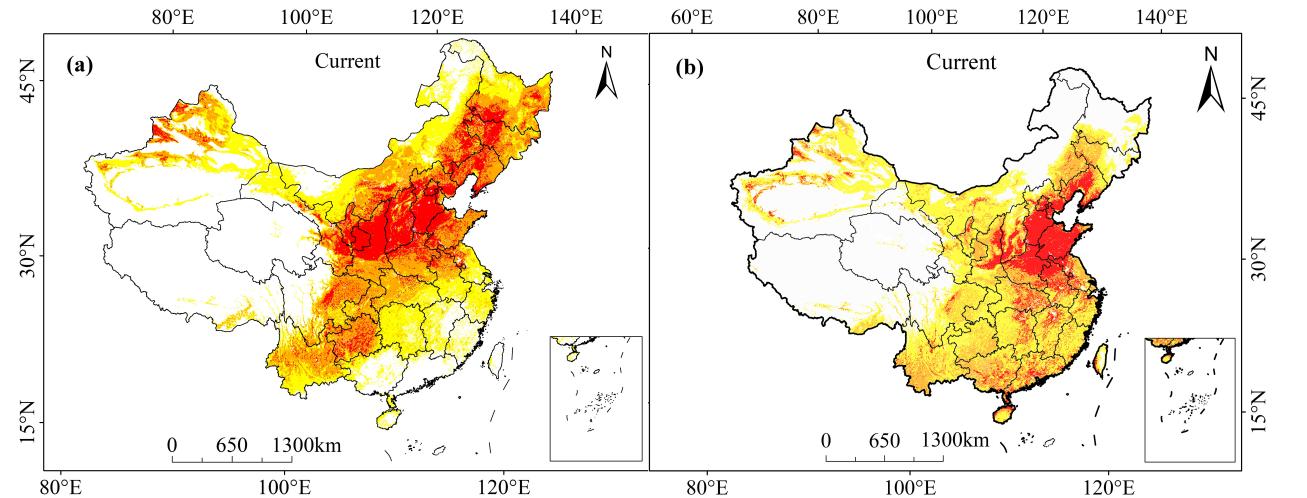


Fig. S.4. Current potential suitable areas of the host plants based on MaxEnt. (a) *H*. *annuus*; (b) *S*. *lycopersicum*


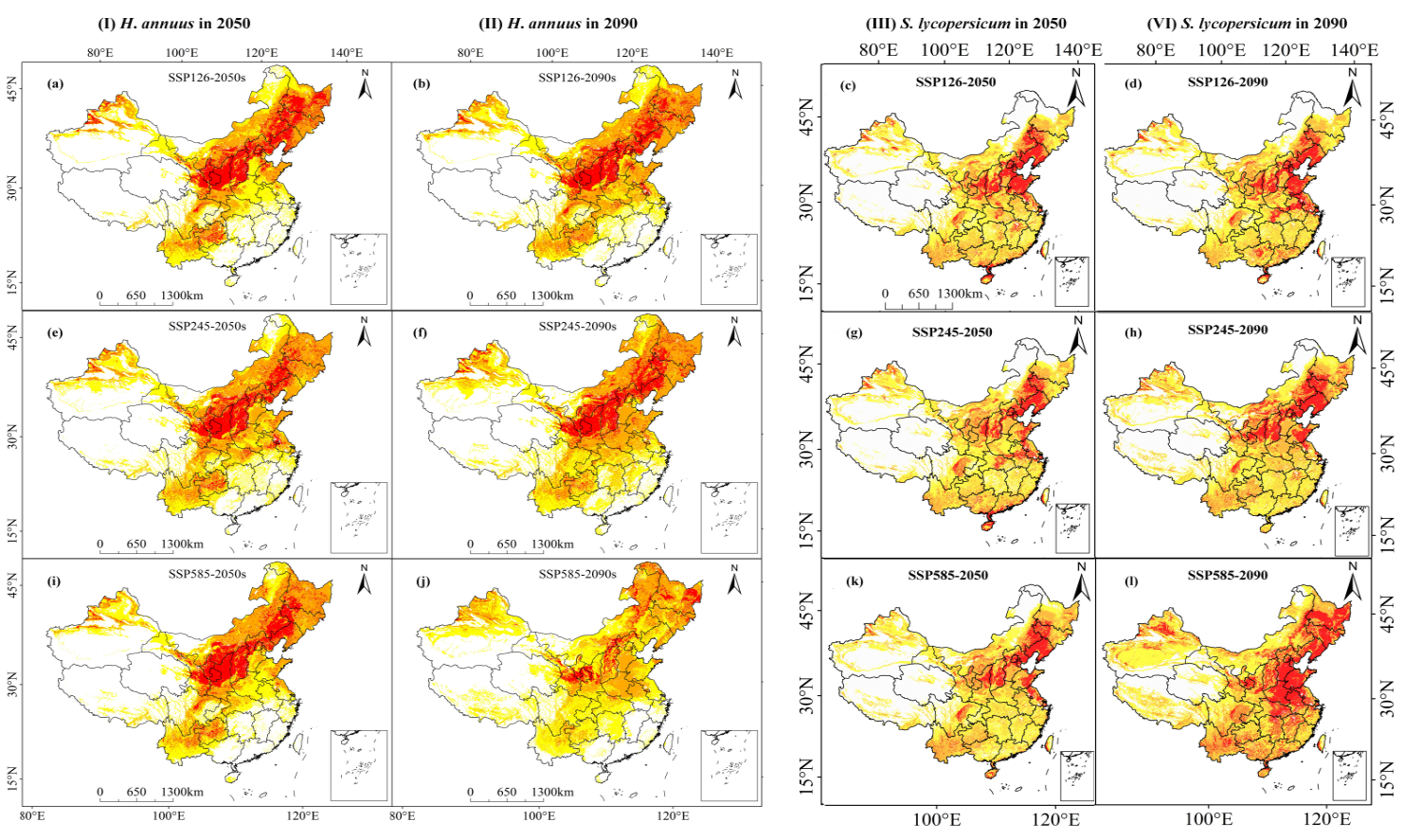


Fig. S.5. Prediction of suitable future suitable areas of *H*. *annuus* and *S*. *lycopersicum* in various SSPs.


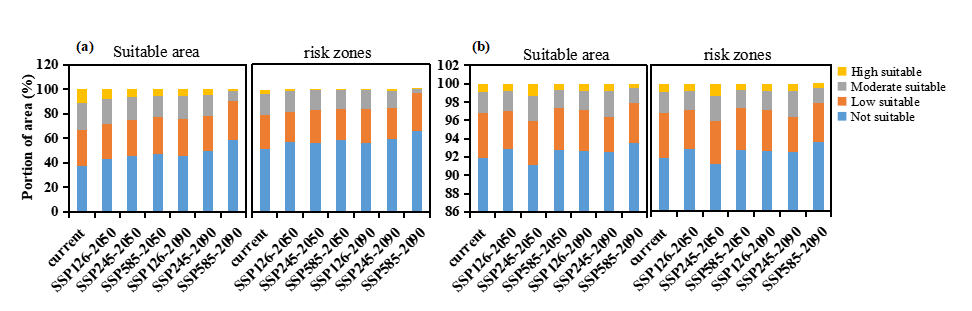
Fig. S.6. The suitable area and risk zones proportion of *O. cumana* and *P. aegyptiaca* under future climate scenarios. (a) *O. cumana*; (b) *P. aegyptiaca*.

**(a)**
